# Programmed cell death-related prognostic genes mediate dysregulation of the immune microenvironment in triple-negative breast cancer

**DOI:** 10.3389/fimmu.2025.1563630

**Published:** 2025-03-12

**Authors:** Xiaowen Ma, Hui Shan, Zhao Chen, Rongzi Shao, Ning Han

**Affiliations:** ^1^ Pharmacy Department, 960th Hospital of the Joint Logistic Support Force, Jinan, Shandong, China; ^2^ Department of Clinical Laboratory, Qilu Hospital of Shandong University (Qingdao), Qingdao, Shandong, China; ^3^ Thoracic Surgery Department, 960th Hospital of the Joint Logistic Support Force, Jinan, Shandong, China; ^4^ Department of Clinical Laboratory, 960th Hospital of the Joint Logistic Support Force, Jinan, Shandong, China

**Keywords:** programmed cell death, triple-negative breast cancer, immune microenvironment, prognostic model, fibroblasts, drug resistance

## Abstract

**Background:**

Programmed Cell death (PCD) encompasses a spectrum of genetically regulated cell death processes and plays a double-edged sword role in neoplastic progression and therapeutic resistance of Triple-Negative Breast Cancer(TNBC)through the tumor microenvironment (TME). However, the specific mechanisms by which PCD mediates microenvironmental dysregulation remain elusive.

**Methods:**

Analyzing nine samples of TNBC through single-cell RNA sequencing (scRNA-seq), this study employed nonnegative matrix factorization (NMF) to assess genes associated with 13 PCD modes. Single-cell regulatory network inference and clustering (SCENIC), Monocle, CellChat, and scMetabolism were used for pseudotime analysis, intercellular communication mapping, determination of transcription factor activities (TFs), and immune infiltration of PCD-related cell clusters in TME. A robust prognostic model and drug resistance analysis were constructed using gene set enrichment analysis (GSEA), Kaplan-Meier survival analysis, and multivariable Cox regression. Finally, hub genes and critical PCD-related cell clusters were validated in the clinical breast cancer samples and the TNBC model mice.

**Results:**

This investigation demonstrated that PCD significantly modulated the functional and phenotypic diversity of fibroblasts, macrophages, T cells, and B cells in the TME of TNBC. Furthermore, this study revealed that PCD-regulated CEBPB-positive cancer-associated fibroblast (CAF) populations are a key determinant of the TNBC immune Microenvironment heterogeneity and poor prognosis. Notably, CellChat analysis unveiled diverse and extensive interactions between PCD-related cell clusters and tumor immune cells, highlighting the CEBPB+ CAF subtype as a signaling ligand communicated with other immune cell clusters through the Midkine (MDK)-Nucleolin (NCL) signaling axis. Moreover, the TIDE analysis verified that CEBPB+ CAF is a predictor of poor prognosis in Immunotherapy. The ex vivo analyses of tumor specimens from both TNBC patients and syngeneic murine models were performed by quantitative reverse-transcription PCR (qRT-PCR), immunoblotting, immunohistochemical staining, and multiplexed immunofluorescence co-localization assays. They confirmed differential expression of the PCD-related prognostic genes and the presence of CEBPB+ CAFs.

**Conclusion:**

In summary, our study provides a comprehensive molecular framework to understand the role of PCD-mediated TME dysregulation in TNBC pathogenesis. This study also offers new insights into the underlying mechanisms of immune therapy resistance in TNBC and identifies promising therapeutic targets for enhancing treatment efficacy and patient outcomes.

## 1 Introduction

Breast cancer (BC) is one of the most common malignant tumors among women. It accounts for approximately 31% of new cancer cases and is the second leading cause of cancer-related deaths among women Triple-negative breast cancer (TNBC) demonstrates significantly higher invasive and metastatic abilities than the other types of BC. The high recurrence rates of TNBC are associated with its increased propensity for vascular invasion. Therefore, patients with TNBC have the poorest prognosis among the various BC types (1). Furthermore, because of the lack of ER, PR, and HER2 expression, the efficacy of conventional treatments such as endocrine and targeted therapies is limited, and advanced patients with TNBC have a median survival of less than 24 months (2). In recent years, immunotherapy targeting the tumor microenvironment (TME) has shown higher clinical efficacy in patients with TNBC. The cell types within the TME of TNBC patients play contradictory roles in tumor growth and progression. Some cell types promote TNBC progression by secreting and expressing factors that enhance tumor cell proliferation and suppress anti-tumor immune responses. However, other cell types in the TME of TNBC patients suppress tumor growth by promoting adaptive immunity (1, 3). In the phase I KEYNOTE-012 clinical trial, PD-1 inhibition via pembrolizumab monotherapy demonstrated sustained antineoplastic activity in both early-stage and advanced PD-L1-positive TNBC patients (Combined Positive Score [CPS] ≥ 1), but a subset of TNBC patients exhibited primary resistance to this immunotherapeutic intervention (4). Zhang et al. stratified TNBC into two distinct immunological subtypes, namely, macrophage-enrichment subtype and neutrophil-enrichment subtype, and identified differential mechanisms of immunotherapy resistance across myeloid cell populations in TNBC (5). The underlying mechanisms of immunotherapeutic resistance in TNBC are complex because of significant TME heterogeneity and intricate cellular crosstalk between the neoplastic cells and the diverse stromal cell populations within the TME. Consequently, an in-depth analysis of the TME-mediated immunotherapy resistance mechanisms is necessary to improve the therapeutic outcomes and enhance the prognostic indicators for TNBC patients.

Immunological heterogeneity within the tumor microenvironment is associated with differential outcomes of treatment modalities targeting the programmed cell death (PCD) mechanisms in the cancer cells (6). PCD encompasses a spectrum of genetically regulated cell death processes that are orchestrated by distinct molecular cascades and signal transduction pathways. PCD plays a pivotal role in neoplastic progression, therapeutic resistance, and immunological escape mechanisms. It is currently known that the PCD spectrum comprises 13 distinct modalities, including macroautophagy, type I PCD (apoptosis), RIPK1/RIPK3-mediated necroptosis, inflammatory caspase-dependent pyroptosis, copper-induced cuproptosis, and iron-dependent ferroptosis (7, 8). Neoplastic cells undergoing multimodal PCD modulate the immune system by secreting a wide array of cytokines, which facilitate chemotactic recruitment of diverse immune cell populations and phenotypic transformation of tumor-infiltrating lymphocytes and resident immune cells into immunosuppressive phenotypes. This facilitates TME remodeling and promotes immune evasion and therapeutic refractoriness (9). Conversely, PCD mechanisms facilitate conventional dendritic cell (cDC) trafficking to the tumor-draining lymph nodes (tdLNs) and potentiate adaptive immune responses by releasing damage-associated molecular patterns and proinflammatory mediators, thereby suppressing tumor growth and enhancing immunotherapeutic efficacy (10). This functional dichotomy has established PCD as a critical homeostatic regulator within the TME. Therefore, PCD has emerged as a central focus in immuno-oncological research. Previous studies have reported that PCD mechanisms are dysregulated in TNBC (11). Novel pharmacological agents targeting the PCD pathways have demonstrated significant therapeutic potential in TNBC. Chen et al. reported that phloretin (Ph), a dihydrochalcone flavonoid derivative, suppressed the proliferation of TNBC cells by downregulating the mTOR/ULK1 signaling pathway and suppressing autophagy (12). Spirooxindole 6e induced PCD in the MDA-MB-231 cells by modulating the intrinsic apoptotic pathway through downregulation of Bcl-2, upregulation of Bax, and activation of caspase-3 (13). The current research paradigms focus on individual PCD mechanisms in TNBC. However, sustained modulation of individual PCD pathways may induce therapeutic resistance. Furthermore, the bidirectional effects of PCD represent significant challenges in developing precision-targeted therapeutics for TNBC (8). The exponential expansion of public genomic repositories and revolutionary advances in single-cell analytical methods have facilitated multi-omics approaches to identify novel tumor pathogenetic mechanisms and therapeutic targets.

This study comprehensively analyzed data derived from Next Generation Sequencing to unravel novel cellular phenotypes and molecular signatures mediated by PCD-related prognostic genes in TME of TNBC. Applying nonnegative matrix factorization (NMF), single-cell regulatory network inference and clustering (SCENIC), gene set enrichment analysis (GSEA) and CellChat, this study aimed to explore signaling pathways, functional enrichments, intricate interplay, transcriptional features, immune characteristics, various developmental roles and prognostic implications within these distinct subgroups of CAFs, macrophages, T cells, and B cells in the TME, providing a robust framework for developing highly effective targeted therapeutics for TNBC. In summary, this study sheds light on the potential role of PCD-related prognostic genes in the dysregulated immune microenvironment in Triple-Negative Breast Cancer, and provides a robust framework for developing highly effective targeted therapeutics for TNBC.

## 2 Materials and methods

### 2.1 Single-cell data processing

We downloaded the single-cell RNA sequencing dataset GSE176078 (14)from the Gene Expression Omnibus (GEO) database (www.ncbi.nlm.nih.gov/geo) and extracted nine single-cell samples of triple-negative breast cancer (TNBC). We calculated and filtered cells that expressed more than 300 genes using the PercentageFeatureSet function, with mitochondrial gene expression below 15%, and red blood cell gene proportion less than 1%. The merged ScRNA-seq data were normalized, and the top 2000 highly variable genes were identified using the FindVariableFeatures function. We then dimensionality reduction on the selected top 2000 highly variable genes using the ScaleData function and the RunPCA function. Batch correction was performed using the Harmony algorithm. Cells were clustered using the ‘FindNeighbors’ and ‘FindClusters’ functions (resolution=0.3) to identify cell clusters, and further dimensionality reduction was carried out using the UMAP method. Finally, the FindAllMarkers function was used to screen for marker genes in six subgroups, which were annotated and visualized using references and the CellMarker 2.0 database (15). After the inferCNV package was employed for tumor cell identification, we used the Monocle2 package (16) for pseudotime analysis of the subgroups.

Additionally, we obtained transcriptome data of 121 TNBC patients with survival information and normal patients from the University of California Santa Cruz (UCSC) Xena Browser (https://xenabrowser.net/). We also downloaded datasets GSE58812 and GSE21653 from the GEO database for subsequent transcriptome-level validation. All data required for this study are publicly accessible, and we provide all the codes in the attached materials (**Supplementary Code Data Package**). Differential gene expression between TNBC and normal groups was calculated using the limma package (17), with a selection criterion of p-value < 0.05.

### 2.2 Programmed death prognostic gene selection

The programmed death gene set encompasses 13 distinct modes of cell death (18), including apoptosis, pyroptosis, ferroptosis, autophagy, entotic cell death, cuproptosis, parthanatos, netotic cell death, alkaliptosis, lysosome-dependent cell death, and oxeiptosis, as well as disulfidptosis (**Supplementary Table S1**). We intersected this gene set with the differentially expressed genes (DEGs) identified in triple-negative breast cancer (TNBC). Subsequently, a univariate Cox regression analysis was performed on the intersected genes, resulting in the selection of 37 prognostic genes with a p-value < 0.05 for further analysis (**Supplementary Table S2**).

### 2.3 Programmed death gene non-negative matrix factorization

We employed non-negative matrix factorization (NMF) to decompose the gene expression matrix and extract biologically meaningful patterns (19). To explore the effects of selected programmed death genes on the tumor microenvironment (TME), we specifically extracted cells from tumor patients. We then applied dimensionality reduction using the NMF R package (version 0.20.6) to analyze the expression of 37 prognostic genes associated with programmed death within the TME. Following this analysis, distinct cell subtypes were identified based on the single-cell RNA (scRNA) expression matrix.

### 2.4 Identification of programmed death gene cell subtypes in the tumor microenvironment

We utilized the FindAllMarkers function to identify marker genes associated with each non-negative matrix factorization (NMF) subtype within the TME. The NMF-derived cell subgroups were characterized based on the following criteria: (1) marker genes exhibited an absolute log2 fold change (Log2FC) greater than 1 and a p-value less than 0.05; (2) each subgroup was named after the gene with the highest Log2FC among the 37 programmed death genes; if no gene met the criteria within an NMF subgroup, it was designated as ‘none’. We then employed the Add ModuleScore function to compute the signature scores of differentially expressed genes (DEGs) for these NMF subgroups. The FeaturePlot function was applied to visualize the distribution of these DEGs. A list of marker genes for each cluster is provided in **Supplementary Table S3**.

### 2.5 SCENIC analysis for NMF PCD−related subtypes

We utilized the SCENIC package, version R3.1.4, to dissect the regulatory network of transcription factors (TFs) within Triple-Negative Breast Cancer. For enrichment analysis, we leveraged gene-motif rankings from the RcisTarget database, focusing on the hg19-tss-centered-10kb track, to elucidate the regulatory network linked to transcription start sites (TSS) in TNBC. Visualization of the derived regulatory networks was facilitated by the pheatmap package.

### 2.6 Cell–cell communication analysis for PCD−related subtypes

CellChat enables the quantitative inference and analysis of intercellular communication networks from single-cell RNA sequencing (scRNA-seq) data (20). We utilized CellChat to assess the communication processes between all cells and tumor cells within the tumor microenvironment (TME), as well as the interactions between PCD-related cell clusters and malignant epithelial cell subpopulations. The strengths of cell-cell communication networks among all NMF clusters were visualized using the netVisualCircle function.

### 2.7 Functional enrichment analysis of NMF PCD-related subtypes

We utilized the cluster Profiler R package (21) to perform Gene Ontology (GO) annotation and Genes and Genomes (KEGG) pathway enrichment analysis on differentially expressed genes, considering a false discovery rate (FDR) threshold of p < 0.05 as statistically significant. Moreover, we employed the scMetabolism package (22) to assess the metabolic activity of macrophage NMF subtypes, encompassing 85 KEGG pathways and 82 REACTOME metabolic pathways. Additionally, we used the PROGENy R package (23)to calculate the activity of 13 cancer-related pathways (EGFR, MAPK, WNT, PI3K, VEGF, JAK-STAT, TGFβ, TNFα, NFκB, Hypoxia, Estrogen, p53, and Trail) across each NMF PCD-related subtype.

### 2.8 Survival analysis of NMF PCD-related subtypes

We classified the NMF PCD-related subtypes based on marker genes identified using the FindAllMarkers function in the TCGA database. Meanwhile, we conducted univariate Cox proportional hazards regression analysis to identify subtypes with prognostic significance. Subsequently, the survminer R package was employed to generate survival curves for the high- and low-risk groups stratified by these subtypes. Consistent with this approach, we also processed the GSE58812 and GSE21653 datasets.

### 2.9 Selection and model construction of prognostic genes in clusters

We identified PCD-related clusters that exhibit high expression in tumors and possess prognostic significance, selecting one particular cluster for further analysis. We determined the prognostic genes by intersecting the markers of this cluster with the differentially expressed genes between TNBC and normal tissues, followed by univariate Cox regression analysis of genes with a p-value < 0.05, resulting in 14 identified genes (**Supplementary Table S4**).

To develop a consensus Immune-Related Landscape Score (IRLS) with high precision and reliability, we integrated 10 machine learning algorithms to form 97 distinct algorithmic combinations. The algorithms included Random Survival Forest (RSF), Elastic Net (Enet), Lasso, Ridge, Stepwise Cox, CoxBoost, Partial Least Squares Regression for Cox (plsRcox), Supervised Principal Component (SuperPC), and Support Vector Machine for survival (survival-SVM). The signature generation process included the following steps: (a) Univariate Cox regression identified differential genes in the TCGA-TNBC cohort; (b) We then applied 101 algorithmic combinations to fit predictive models within leave-one-out cross-validation (LOOCV) framework specific to the TCGA-TNBC cohort; (c) Each model was independently validated using two external datasets (GSE58812, GSE21653); (d) For each model, Harrell’s Concordance Index (C-index) was calculated across all validation datasets, and the model demonstrating the highest average C-index was deemed optimal.

### 2.10 Association of key genes with immunity and pathways

We utilized the ESTIMATE algorithm to assess the levels of stromal and immune cells in malignant tumor tissues based on expression data. The algorithm, obtained from the public website (https://sourceforge.net/projects/estimateproject/), estimates stromal and immune scores based on specific biomarkers associated with stromal and immune cell infiltration in tumor samples. These scores were analyzed separately to predict the levels of stromal and immune cells in tumor tissues. We calculated the StromalScore and ImmuneScore for each sample and then determined the correlation of key genes with these scores.

We employed the MCP-counter method (24), which robustly quantifies the absolute abundance of eight immune cell types and two stromal cell populations from transcriptome data (T cells, CD8T cells, Cytotoxic lymphocytes, B lineage, NK cells, Monocytic lineage, Myeloid dendritic cells, Neutrophils). Subsequently, we calculated the correlation of key genes with these populations.

Then, we used the ssGSEA method from the Gene Set Variation Analysis (GSVA) R package to identify genes associated with 28 immune cell types, as reported in the literature (25).

Additionally, we obtained the 50 HALLMARK pathways from the h.all.v7.5.symbols.gmt file at the GSEA website and used the ssGSEA method to calculate pathway scores for samples. We fianally determined the correlation of key genes with these pathways.

### 2.11 Cell culture

The MDA-MB-231 (SCSP-5043) and 4T1 (SCSP-5056) were purchased from the National Collection of Authenticated Cell Cultures. All cell lines were cultured in appropriate culture media supplemented with 1% Penicillin/Streptomycin and 10% fetal bovine serum (Gibco), and maintained at 37 °C and 5% CO2. The cell lines have been STR-authenticated and were routinely tested for mycoplasma-free.

### 2.12 Animal study

Female BALB/c mice (6 weeks old) were purchased from Pengyue Experimental Animal Breeding Co, LTD (Jinan, China) and bred in animal facilities under specific pathogen-free conditions. All mouse procedures and experiments for this study were approved by the Animal Care and Utilization Committee of 960 Hospital. 4T1 cells in the exponential growth phase were digested with trypsin and washed three times with cold PBS. After centrifugation, cells were resuspended in cold PBS. 100 μl cold PBS containing 1×10^6^ 4T1 cells was orthotopically inoculated on the right flank of the fourth pair of mammary fat pads of mice. Tumor growth was monitored with a caliper and volume was calculated as (length × width^2^)/2. According to the experimental plan, tumor cells were sampled on the 7th, 14^th^, and 21st days after injection, all tumor volumes did not exceed 1500 mm^3^.

### 2.13 RT-PCR assay

Total RNA was extracted by using the PARKeasy Tissue/Cell RNA Rapid Extraction Kit (SPARKjade, AC0202, Shandong, China). The cDNA was synthesized using a cDNA synthesis kit (RR037A; Takara, Kyoto, Japan). The primers (Shanghai Bioengineering, Stable 1.), cDNA, TB Green^®^ Premix Ex Taq™ II (Takara, RR820A, Kyoto, Japan), and non-RNase dH2O were thoroughly mixed and then placed into a real-time PCR detection system, as instructed by the manufacturer. Real-time PCR mixture samples were detected by use of the SLAN-96P Real-Time PCR Detection System (SLAN-96P, Hongshi, Shanghai, China) the thermo-cycling conditions were initiated at 95°C for 30 s, followed by 40 cycles of 95°C for 5 s and 60°C for 30 s. Relative gene expression was calculated using the 2^−ΔΔct^ method.

### 2.14 Western blotting

Breast tumor tissues were frozen in liquid nitrogen and ground into a powder in a mortar. Powdered breast tumor tissues were lysed in RIPA buffer containing protease and phosphatase inhibitors (Selleck), and protein quantification was performed using the BCA Protein Assay kit (Solarbio). Next, proteins were isolated using 10% sodium dodecyl sulphate–polyacrylamide gel electrophoresis (SDS-PAGE gel, Solarbio) and transferred to a 0.45-μm polyvinylidene fluoride (PVDF) membrane (Merck Millipore, Billerica, MA, USA). After blocking in 5%(M/V) skim milk for 1 h, the samples were incubated with primary antibody at 4°Covernight. The membranes were then incubated with goat anti-rabbit/mouse IgG-HRP (Santa Cruz) at 37°C in 1:5000 dilution for 1h. Active bands were identified using an enhanced chemiluminescence (ECL) kit (Merck Millipore, Billerica, MA, USA).

### 2.15 Immunohistochemistry

Breast tumor tissues were collected and fixed in 4% paraformaldehyde, after which the tissues were then embedded in paraffin. 4μm thick formalin-fixed, paraffin-embedded tissue sections were used to perform immunohistochemical staining. After the deparaffinization and rehydration process, sections were heated in a pressure cooker in Tris-EDTA buffer for antigen retrieval and then were blocked by peroxidase endogenous blocking solution for 15 min and protein block for 1h. Sections were incubated with primary antibodies against CEBPB (proteintech, 23431-1-AP), and α-SMA (proteintech, 14395-1-AP) overnight at 4°C in a humid chamber. After washing with PBS, sections were incubated with HRP-conjugated Goat Anti-Rabbit/Mouse secondary antibody at room temperature for 1h, followed with DAB for 3–5 min under the microscope. Nuclei were counterstained with hematoxylin. Finally, sections were dehydrated in a series of alcohols and mounted in neutral gum. The specimens were observed using a confocal laser scanning microscope (CLSM; Olympus, Tokyo, Japan), and the area of staining was quantified using ImageJ software.

## 3 Results

### 3.1 PCD is associated with the tumor immune microenvironment in TNBC

**Figure 1:**
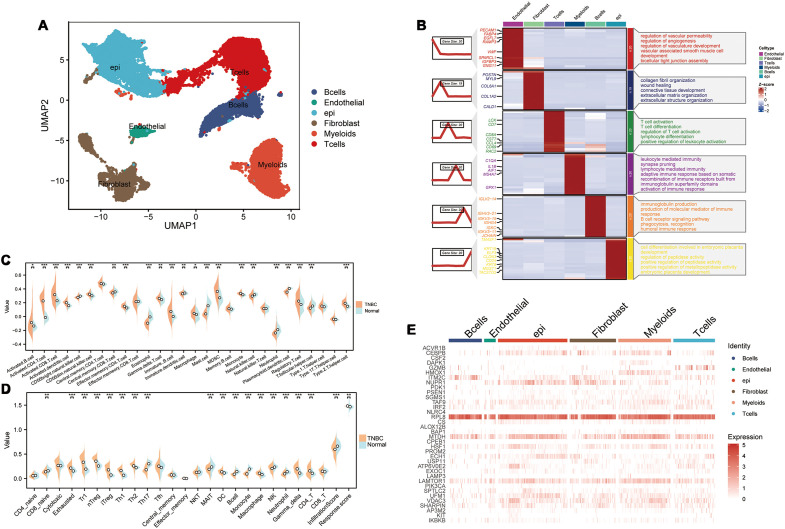
PCD-related prognostic genes in the transcriptome and single-cell landscape of TNBC. **(A)** GSE176078 single-cell UMAP plot; **(B)** Heatmap of marker genes for various cell types; **(C)** ssGSEA analysis comparing immune cell differences between TNBC and Normal; **(D)** ImmuneCellA analysis comparing immune cell differences between TNBC and Normal; **(E)** Expression levels of 37 PCD-related prognostic genes at the single-cell level. *p < 0.05, **p < 0.01, ***p < 0.001.

We performed single-cell RNA sequencing analysis of nine TNBC samples from the GEO TNBC dataset GSE176078. We identified T cells, B cells, myeloid cells, epithelial cells, fibroblasts, and endothelial cells as the main cell types in the TME (**Figure 1A**). Subsequently, the ClusterGVis package was used to visualize the marker genes for each cell type and the GO and KEGG pathway analyses were performed to identify the pathways enriched in each cell type. The results were consistent with the biological functions of the corresponding cell types (**Figure 1B**). Our data showed that all the epithelial cells in the TNBC samples exhibited copy number variations. Therefore, we concluded that the epithelial cells in the TNBC samples were all malignant. This finding was also consistent with their biological characteristics (**Supplementary Figure S1**). Next, to determine the differences in the immune microenvironment between the TNBC and normal tissues, we used the “ssGSEA” and “ImmuneCellA” packages to analyze and compare the immune cell populations in the TME between 121 TNBC patients with survival information and normal patients from the UCSC database (**Supplementary Table S5**). The results demonstrated that several immune cell types, including cytotoxic immune cells such as CD8+ T cells, CD4+ T cells, and NK cells, as well as B cells, macrophages, and fibroblasts were significantly increased in the TNBC tissues compared to the normal breast tissues, thereby indicating significantly altered immune microenvironment in TNBC (**Figures 1C, D**). In the subsequent analyses, we focused primarily on the CD8+T cells, CD4+T cells, NK cells, Tregs, B cells, macrophages, and fibroblasts. We also performed analysis to identify differentially expressed genes between TNBC and normal breast tissues. After intersecting these genes with a set of 13 PCD genes, we performed univariate Cox regression analysis to identify 37 PCD-related prognostic genes (**Supplementary Table S2**). We analyzed the expression levels of these prognostic genes at the single-cell level and found that the 37 PCD-related prognostic genes were highly expressed across various cell types (**Figure 1E**). This suggested that the 37 PCD-related prognostic genes played a key role in multiple biological functions of TNBC.

### 3.2 PCD regulates functional and phenotypic diversity of cell types in the TME

**Figure 2:**
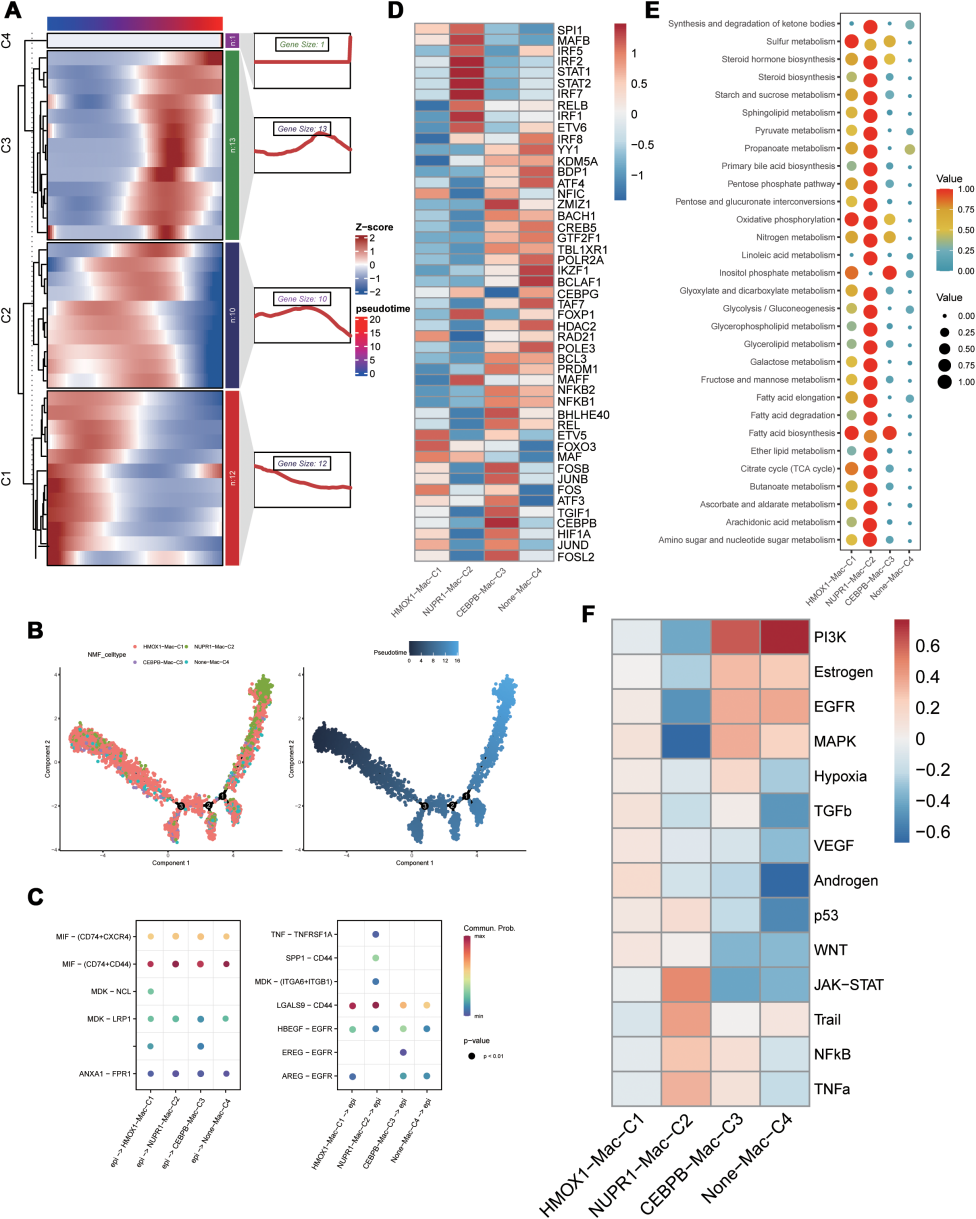
Functional differences of PCD-related genes in macrophages. **(A)** Pseudotime sequence analysis of PCD-related risk genes; **(B)** Pseudotime trajectory analysis of PCD-related clusters; **(C)** Cellular communication between PCD-related clusters and malignant epithelial cells; **(D)** SCENIC analysis of PCD-related clusters; **(E)** Metabolic activity analysis of PCD-related clusters; **(F)** Differential correlation of tumor-related pathways in PCD-related clusters.

We extracted the stromal cells and identified subpopulations of macrophages through dimensionality reduction and annotation. Next, based on the PCD-related prognostic genes, non-NMF clustering was performed and four distinct cell clusters, namely, HMOX1-Mac-C1, NUPR1-Mac-C2, CEBPB-Mac-C3, and Non-Mac-C4, were identified. Pseudotime analysis demonstrated that different PCD-related prognostic genes were differentially expressed during various developmental phases of macrophages. Moreover, the HMOX1-Mac-C1 and Non-Mac-C4 clusters were located at the initial part of the developmental pseudotime trajectory and the NUPR1-Mac-C2 cluster was positioned at the terminal end of the trajectory (**Figures 2A, B**). Cell communication analysis suggested that NUPR1-Mac-C2 acted as a communication receptor and participated in the intercellular communication with the malignant epithelial cells through the MDK-(ITGA6+ITGB1), SPP1-CD44, and TNF-TNFRSF1A pathways (**Figure 2C**). SCENIC analysis identified differences in the transcriptional activity between various clusters. Specifically, the NUPR1-Mac-C2 cluster showed high expression of transcription factors such as STAT1, STAT2, and IRF7, whereas the CEBPB-Mac-C3 cluster showed high expression of transcription factors such as JUND, CEBPB, JUNB, and FOSB (**Figure 2D**). Based on the metabolic analysis, the NUPR1-Mac-C2 cluster exhibited high metabolic activity because of high expression of genes involved the TCA cycle and other pro-tumorigenic pathways (**Figure 2E**), thereby suggesting a close association with tumor growth. Progeny analysis demonstrated associations between the NUPR1-Mac-C2 cluster and pathways such as JAK-STAT, NF-κB, and TNF-α, thereby further substantiating its role in promoting oncogenesis (**Figure 2F**).

**Figure 3:**
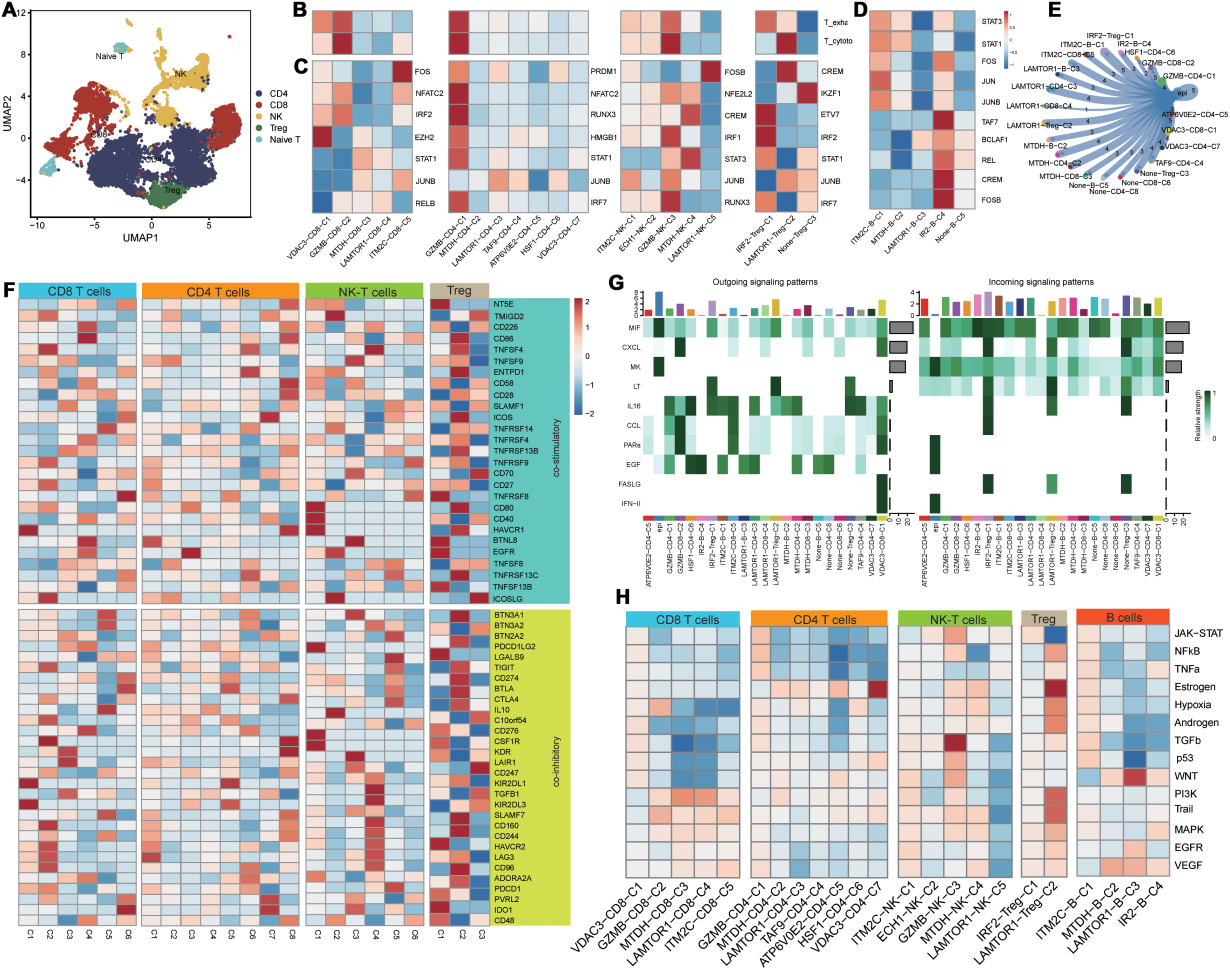
Functional differences of PCD-related T/B cells in the tumor immune microenvironment. **(A)** Dimensionality reduction clustering umap plot of T cells; **(B)** PCD-related T cell clusters in T cell exhaustion and cytotoxicity scoring; **(C)** Transcriptional differences of PCD-related T cell clusters;**(D)** Transcriptional activity differences of PCD-related B cell clusters; **(E)** Communication between PCD-related T/B cell clusters and malignant epithelial cells; **(F)** Expression differences of T cell functional gene sets in PCD-related T cell clusters; **(G)** Heatmap of communication pathways between PCD-related T/B cell clusters and malignant epithelial cells; **(H)** Differences in tumor-related pathway associations of PCD-related T/B cell clusters.

Based on secondary dimensionality reduction and clustering of the immune cells, we identified the following cell types: NK cells, CD8+ T cells, CD4+ T cells, regulatory T cells, naive T cells, and B cells (**Figure 3A**). Subsequently, we performed cell subtyping analysis to assess the functional profiles of each cluster and found that the VDAC3-CD8-C1, GZMB-CD8-C2, and IRF2-Treg-C1 clusters exhibited exhausted T cell phenotype, whereas the GZMB-NK-C3 and LAMTOR1-Treg-C2 clusters displayed cytotoxic phenotype. Furthermore, the GZMB-CD4-C1 cluster demonstrated both cytotoxic and exhausted T cell phenotypes, thereby suggesting functional differences among distinct PCD genes between various cell types (**Figure 3B**). To elucidate differences in the transcriptional activities between these cell clusters, we performed the SCENIC analysis and found that the clusters enriched for the cytotoxic cell or exhausted T cell functions exhibited robust transcriptional activity; among these, GZMB-CD4-C1 cluster showed the highest transcriptional activity (**Figure 3C**). Furthermore, there were differences in the transcription factor profiles between distinct T cell clusters with different functions (**Figure 3C**). Subsequent progeny analysis demonstrated that oncogenic pathways were significantly inhibited in subpopulations of cells with low transcriptional activity (**Figure 3H**). These findings were consistent with the SCENIC analysis. Among the B cells, the IRF2-B-C4 cluster exhibited highest level of transcriptional activity (**Figure 3D**). Cellular communication analysis demonstrated varying degrees of cellular communication between different T and B cell clusters and the epithelial cells (**Figure 3E**). Moreover, functional gene set analysis demonstrated functional differences in T cell activity among various subpopulations (**Figure 3F**). Pseudotime analysis demonstrated that the PCD-related prognostic genes played distinct roles at different time points across various cell types and influenced cluster differentiation (**Supplementary Figure S2**). Cellular communication analysis demonstrated that the immune cell subsets exhibited varied intensities of signaling output, primarily through the IL-6, CCL, and EGF pathways. When the immune cell subsets acted as signaling receptors, the pathways related to MIF and CXCL showed differing strengths, thereby indicating functional disparities (**Figure 3G**).

### 3.3 PCD-regulated CEBPB^+^ CAF subtype is a key determinant of the TNBC Microenvironment heterogeneity and poor prognosis

**Figure 4:**
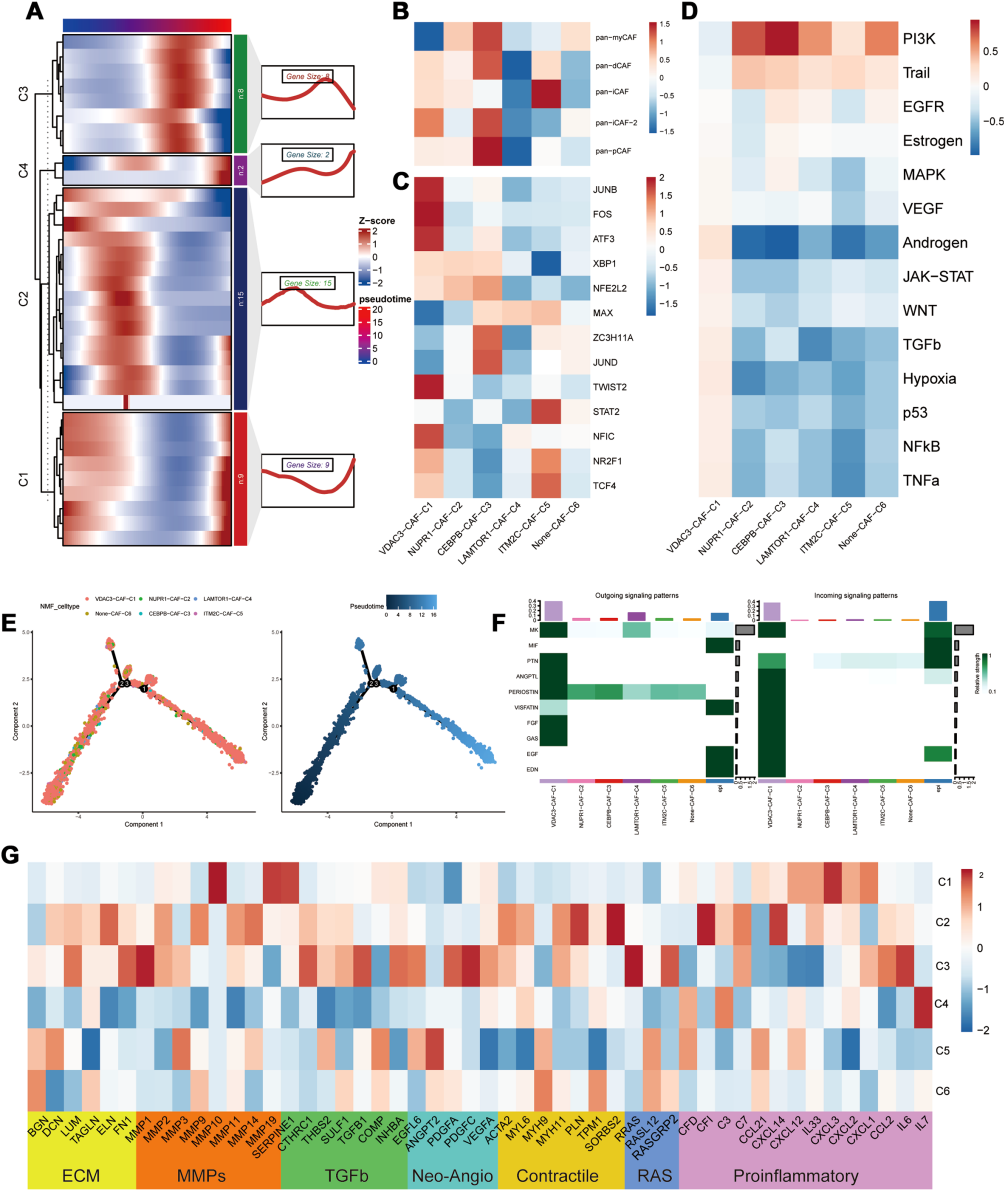
Heterogeneity of PCD-related genes in fibroblasts. **(A)** Distribution of PCD prognostic genes in the pseudo time sequence of PCD-related fibroblasts; **(B)** Typing of PCD-related fibroblasts; **(C)** Differential analysis of transcription factors in PCD-related fibroblasts; **(D)** Analysis of the correlation between PCD-related fibroblasts and oncogenic pathways; **(E)** Differentiation trajectory of PCD-related fibroblast clusters; **(F)** Cellular communication between PCD-related fibroblasts and malignant epithelial cells; **(G)** Differential expression in the tumor microenvironment (TME) of PCD-related fibroblasts.

Pseudotime analysis results demonstrated that different PCD-related prognostic genes exerted their effects in the fibroblasts at distinct time points (**Figure 4A**). Subsequent NMF clustering categorized fibroblasts into the following six clusters: VDAC3-CAF-C1, NUPR1-CAF-C2, CEBPB-CAF-C3, LAMTOR1-CAF-C4, ITM2C-CAF-C5, and Non-CAF-C6. According to pseudotime analysis, VDAC3-CAF-C1 represented a stable subpopulation of fibroblasts throughout the developmental and differentiation process; Non-CAF-C6 cluster was positioned at the initiation of development; and CEBPB-CAF-C3 was located at the midpoint and represented a cluster that differentiated toward subsequent diverse subpopulations (**Figure 4E**). These results suggested a regulatory role for the PCD-related prognostic genes during fibroblast development and differentiation (**Figure 4E**).

To further elucidate the roles of different subpopulations, we assessed the biological functions of each subpopulation of fibroblasts (27). CEBPB-CAF-C3 exhibited biological functions associated with both pan-pCAF and pan-myCAF gene signatures that were characterized by genes related with smooth muscle generation and vascular wound healing, as well as, pan-dCAF and pan-iCAF-2 gene signatures that were related with genes related with the inflammation-related pathways and extracellular matrix (ECM) remodeling. The central location of CEBPB-CAF-C3 in the developmental branch also explains its diverse biological functions. And the CEBPB-CAF-C3 cluster was enriched in transcription factors such as JUND and ZC3H11A (**Figure 4C**). In contrast, VDAC3-CAF-C1 and ITM2C-CAF-C5 were significantly enriched in pathways associated with tumor-related inflammatory responses (**Figure 4B**). The SCENIC analysis demonstrated that the VDAC3-CAF-C1 cluster was enriched in transcription factors such as JUND, FOS, ATF3, and other transcription factors associated with the inflammatory pathways and was consistent with the classification. Further progeny analysis demonstrated that the CEBPB-CAF-C3 cluster was significantly associated with the PI3K-AKT pathway. This suggested that the CEBPB-CAF-C3 cluster may play a role in the occurrence and development of tumors through the PI3K-AKT pathway (**Figure 4D**). To further analyze the relationships between different clusters, we used the cell communication analysis and found that VDAC3-CAF-C1 was closely associated with malignant epithelial cells and various pro-oncogenic pathways (**Figure 4F**). Subsequently, based on the genes related to the tumor microenvironment (TME), we performed functional enrichment analysis of PCD-related fibroblasts. Genes associated with ECM, MMPs, TGF-β, and proinflammatory pathways showed significantly high expression in the CEBPB-CAF-C3 cluster, whereas proinflammatory-related genes were highly expressed in the VDAC3-CAF-C1 cluster (**Figure 4G**). These results aligned accurately with the classification of the CAF subtypes.

**Figure 5:**
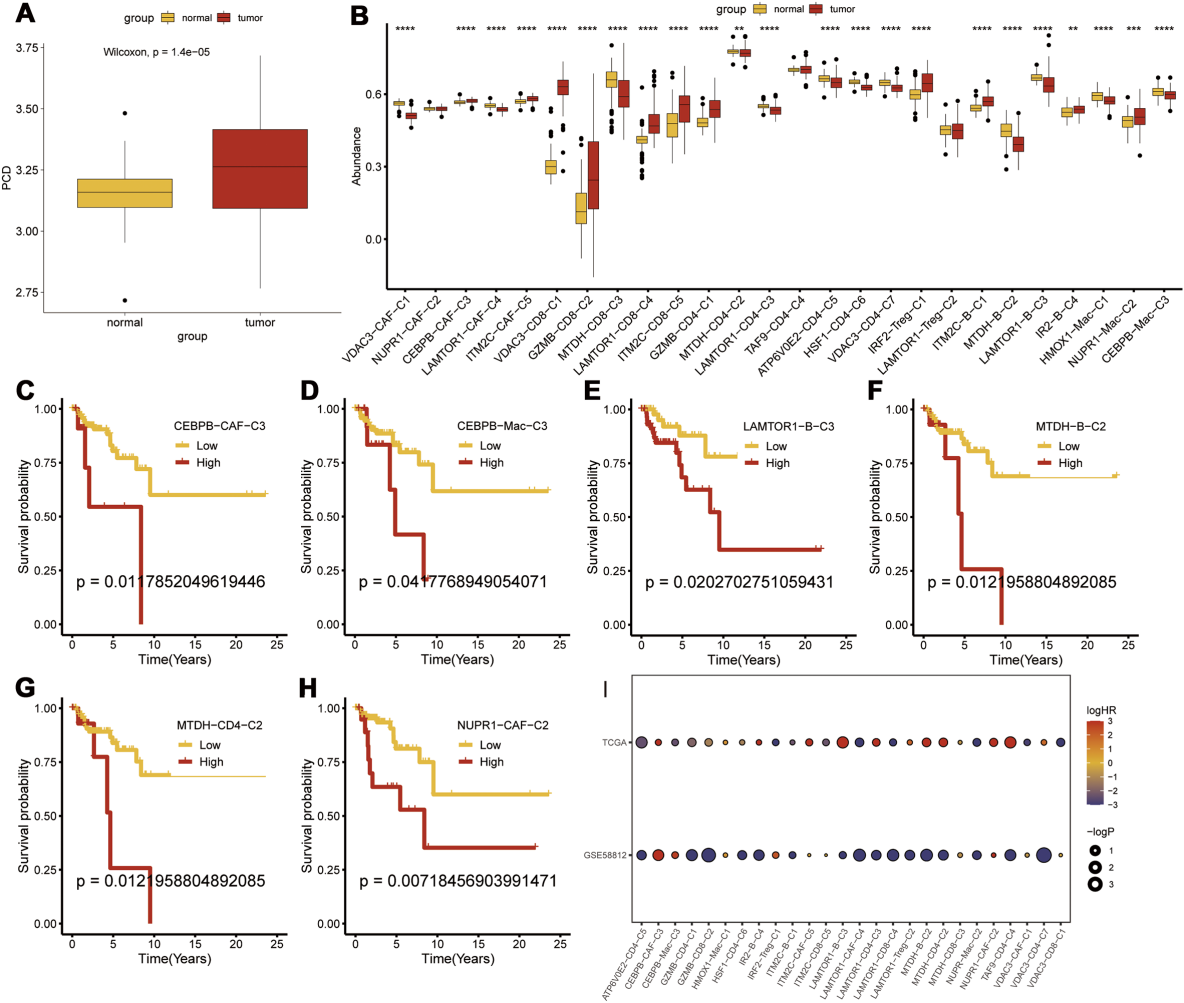
Transcriptomic Prognostic Analysis of PCD-Related Clusters. **(A)** Differential expression of PCD-related prognostic genes between tumor and normal tissues; **(B)** Differential expression of various PCD-related clusters at the transcriptomic level; **(C-H)** Prognostic analysis of subgroups; **(I)** Validation of risk factors for each subgroup in the GSE58812 dataset. p < 0.05, **p < 0.01, ***p < 0.001, ****p < 0.0001.

Transcriptomic analysis was performed to determine the prognostic significance of the PCD-related clusters. First, we validated the differential expression of PCD-related prognostic genes between the TNBC and normal breast tissues and found that the prognostic genes were significantly upregulated in the TNBC tissues (p < 0.001) (**Figure 5A**). We then performed Gene Set Variation Analysis (GSVA) using marker genes for each cluster to compare the transcriptomic differences and found that CEBPB-CAF-C3, IM2C-CAF-C5, VDAC3-CD8-C1, GZMB-CD8-C2, LAMTOR1-CD8-C4, ITM2C-CD8-C5, GZMB-CD4-C1, IRF2-Treg-C1, ITM2C-B-C1, IR2-B-C4, and NUPR1-Mac-C2 were all highly expressed in the TNBC tissues (**Figure 5B**). Prognostic analysis further demonstrated that the differential expression of CEBPB-CAF-C3 exhibited a consistent pattern. This suggested that CEBPB-CAF-C3 potentially represented a key oncogenic subgroup that impacted patient prognosis (**Figures 5C–H**). Further validation in the GSE58812 dataset confirmed that CEBPB-CAF-C3 was associated with poor prognosis and served as a risk factor subgroup (**Figure 5I**).

**Figure 6:**
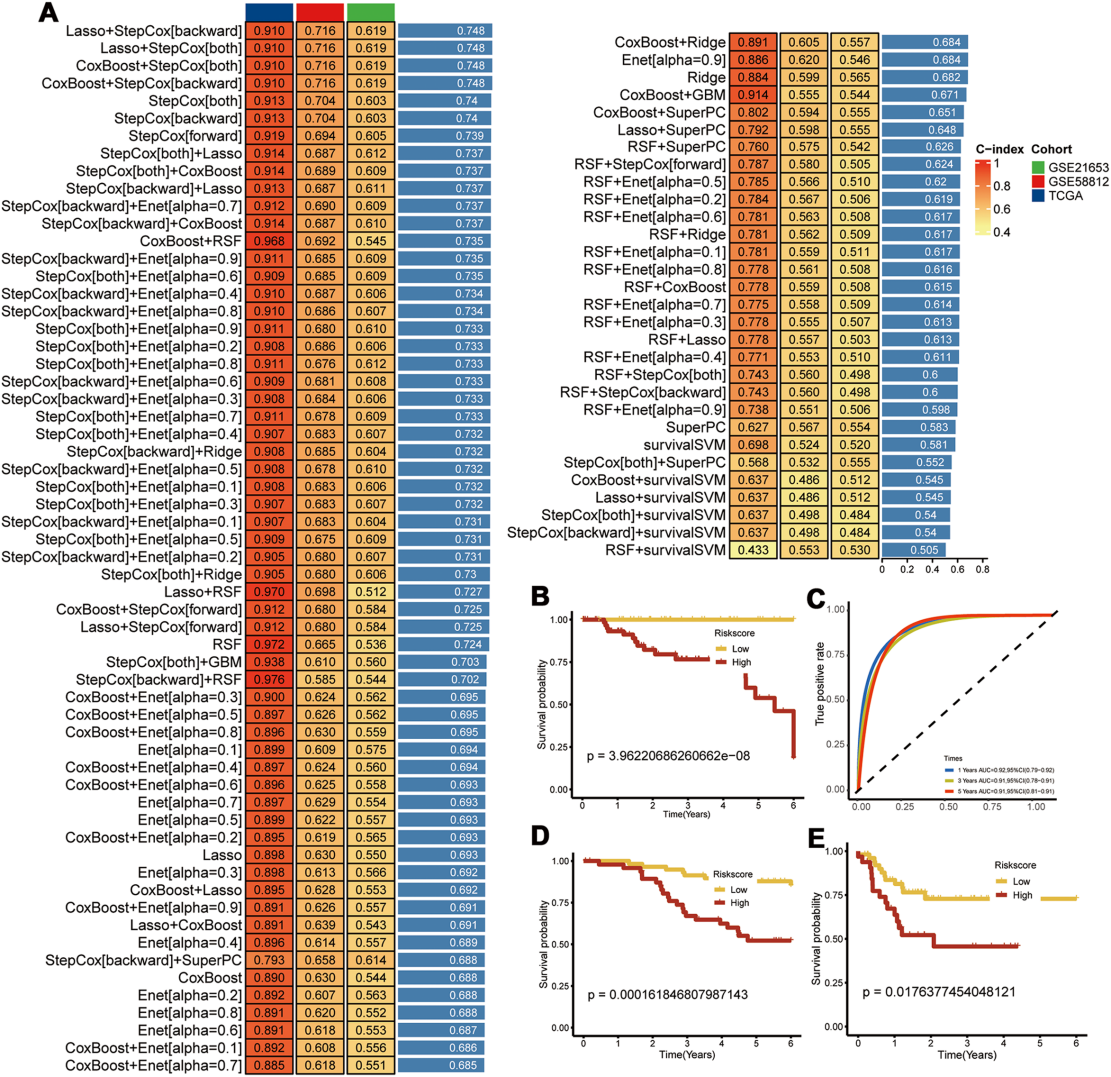
Construction of Prognostic Models. **(A)** Using 97 machine learning algorithms for the construction of prognostic models; **(B)** Prognostic survival analysis in the TCGA-TNBC dataset; **(C)** Area Under the Curve (AUC) analysis; **(D)** Prognostic survival analysis in the GSE58812 dataset; **(E)** Prognostic survival analysis in the GSE21653 dataset.

We identified a set of CEBPB-CAF-C3-specific marker genes and intersected them with the DEGs in the TNBC tissues compared with the normal breast tissues. Univariate Cox regression analysis was used to screen the PCD-related prognostic genes and 14 prognostic genes were identified (**Supplementary Table S4**). A prognostic model was constructed with these genes using an integrative approach based on machine learning. In the TCGA dataset, 97 predictive models were fitted using the Leave-One-Out Cross-Validation method and the concordance index (c-index) was calculated for each model across all the validation datasets (**Figure 6A**).

The model using a combination of Lasso and Stepwise Cox regression with backward elimination was the most optimal with the highest average concordance index (c-index) of 0.748. Among the 14 prognostic genes analyzed, we identified seven key genes, namely, *CEBPB, TANK, MAT2B, TMEM165, CCDC167, CYFIP1*, and *COX17*. Subsequently, the prognostic model was evaluated with the TCGA-TNBC dataset. The survival outcomes were poorer for the high-risk TNBC group compared to the low-risk TNBC group. Moreover, the prognostic model showed excellent prognostic prediction performance with area under the curve values of 0.92, 0.91, and 0.91 for 1-, 3-, and 5-year overall survival, respectively (**Figures 6B, C**). The prognostic model was further validated in the GSE58812 and GSE21653 datasets, which also showed poorer survival outcomes for patients in the high-risk group (**Figures 6D, E**). This demonstrated good predictive power for the prognostic model.

### 3.4 Immunosuppressive mechanism and immunotherapeutic relevance of the CEPBB-CA-C3 subtype in the TME of TNBC

**Figure 7:**
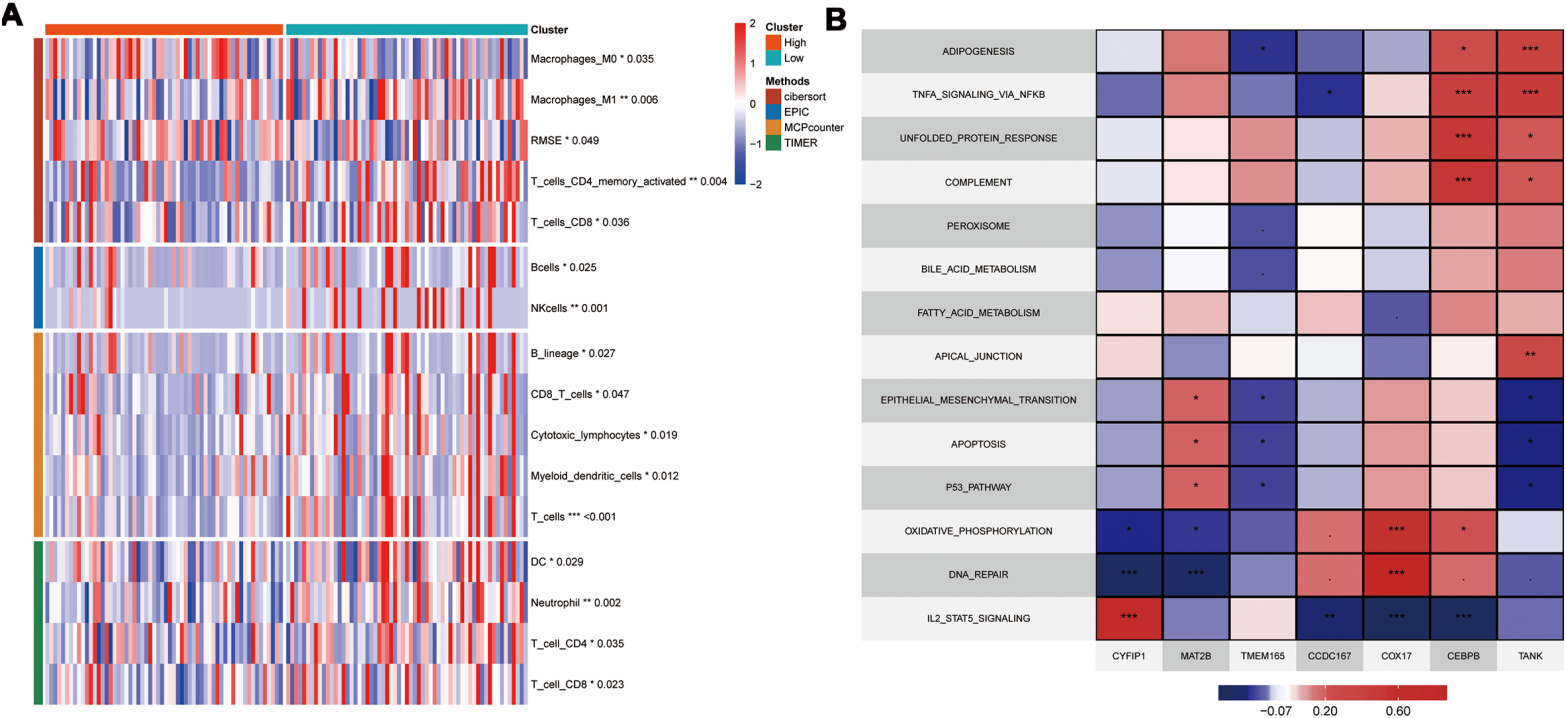
Model and Hub Gene Immune Infiltration Analysis. **(A)** Immune infiltration analysis of model genes; **(B)** Correlation of key genes with pathways in the TCGA; indicates * p<0.05, ** indicates p<0.01, *** indicates p<0.001.

To further elucidate the differences in immune infiltration within the model, we used Cibersort, EPIC, MCPcounter, and Timer algorithms to analyze and assess the prognostic differences between high- and low-risk groups. Our data showed significant immune suppression and depletion of various immune cell types across all four algorithms in the high-risk group compared with the low-risk group (**Figure 7A**). We then assessed the differences in immune infiltration based on the seven hub genes and found that the risk genes (HR > 1) also demonstrated a degree of immune suppression (**Supplementary Figure S3**). Furthermore, we downloaded the HALLMARK pathway files (h.all.v7.5.symbols.gmt) comprising 50 pathways from the GSEA website and used ssGSEA to evaluate the scores of each pathway in the TCGA-TNBC dataset (26). We then calculated the Pearson correlation co-efficients to assess the relationship between the seven key genes and the pathway scores and visualized the results. Our results showed significant differences in the gene function enrichment and the risk genes were enriched in the STAT pathway and TNF-α-related pathways (**Figure 7B**).

**Figure 8:**
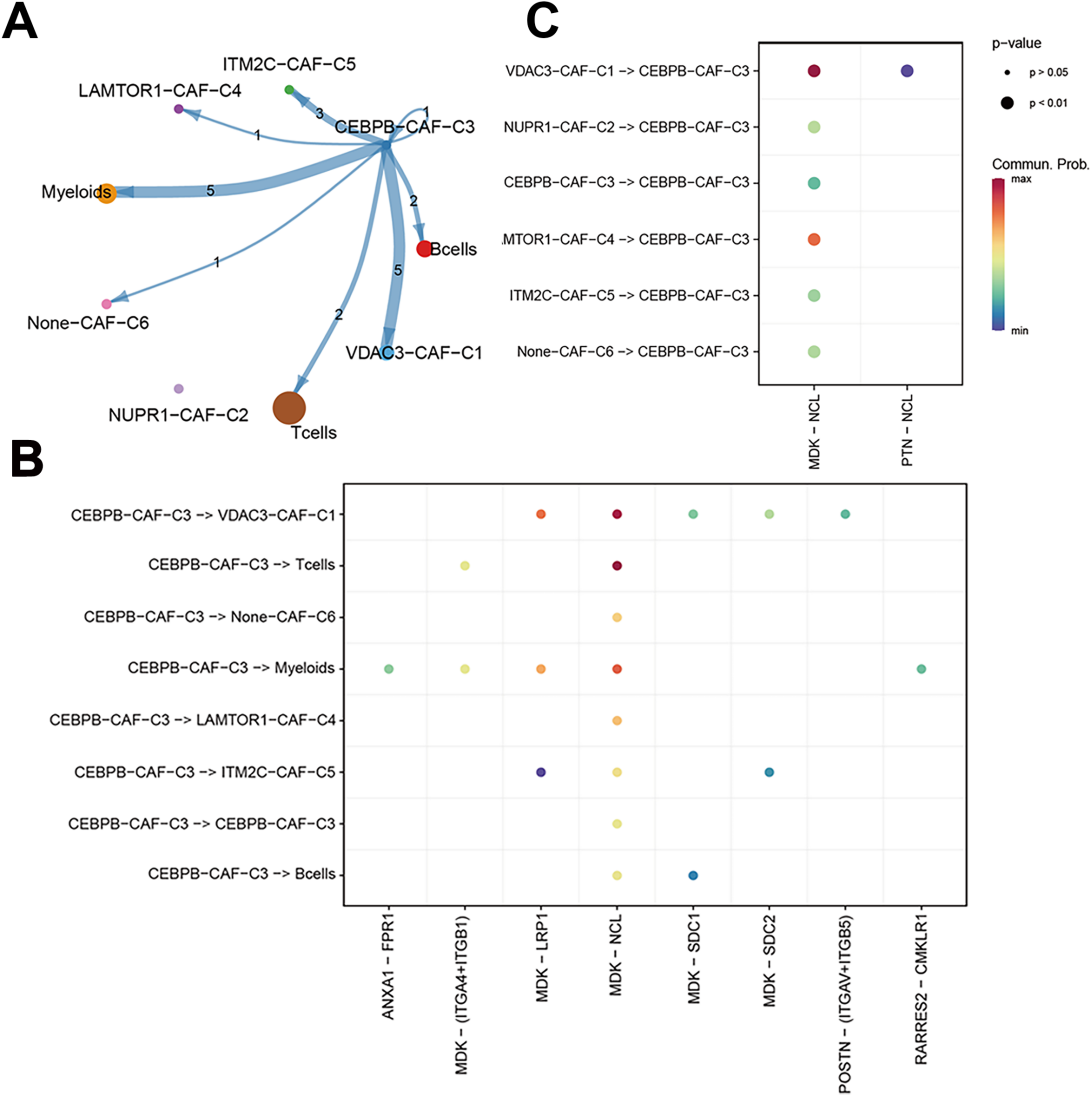
The correlation between CEBPB-CAF-C3 and immune cells. **(A)** Cell communication network diagram; **(B)** Cell communication interaction map with CEBPB-CAF-C3 as a ligand; **(C)** Cell communication interaction map with CEBPB-CAF-C3 as a receptor.

We performed a subtype communication analysis to further investigate the differences in communication between immune cell types (T cells, B cells, and myeloid cells) and the CAF subtypes. The results demonstrated that CEBPB-CAF-C3 communicated with the T cells, B cells, and myeloid cells (**Figure 8A**). Furthermore, CEBPB-CAF-C3 as a signaling ligand communicated with the T cells through the MDK-(ITGA4+ITGB1) and MDK-NCL receptor-ligand pathways, and through MDK-SDC1 receptor-ligand interactions with the B cells, and through multiple pathways, including ANXA1-FPR1, MDK-(ITGA4+ITGB1), MDK-LRP1, MDK-NCL, and RARRES2-CMKLR1 with the myeloid cells (**Figure 8B**). However, when CEBPB-CAF-C3 was used as a signaling receptor, we did not observe any communication with the immune cells (**Figure 8C**).

**Figure 9:**
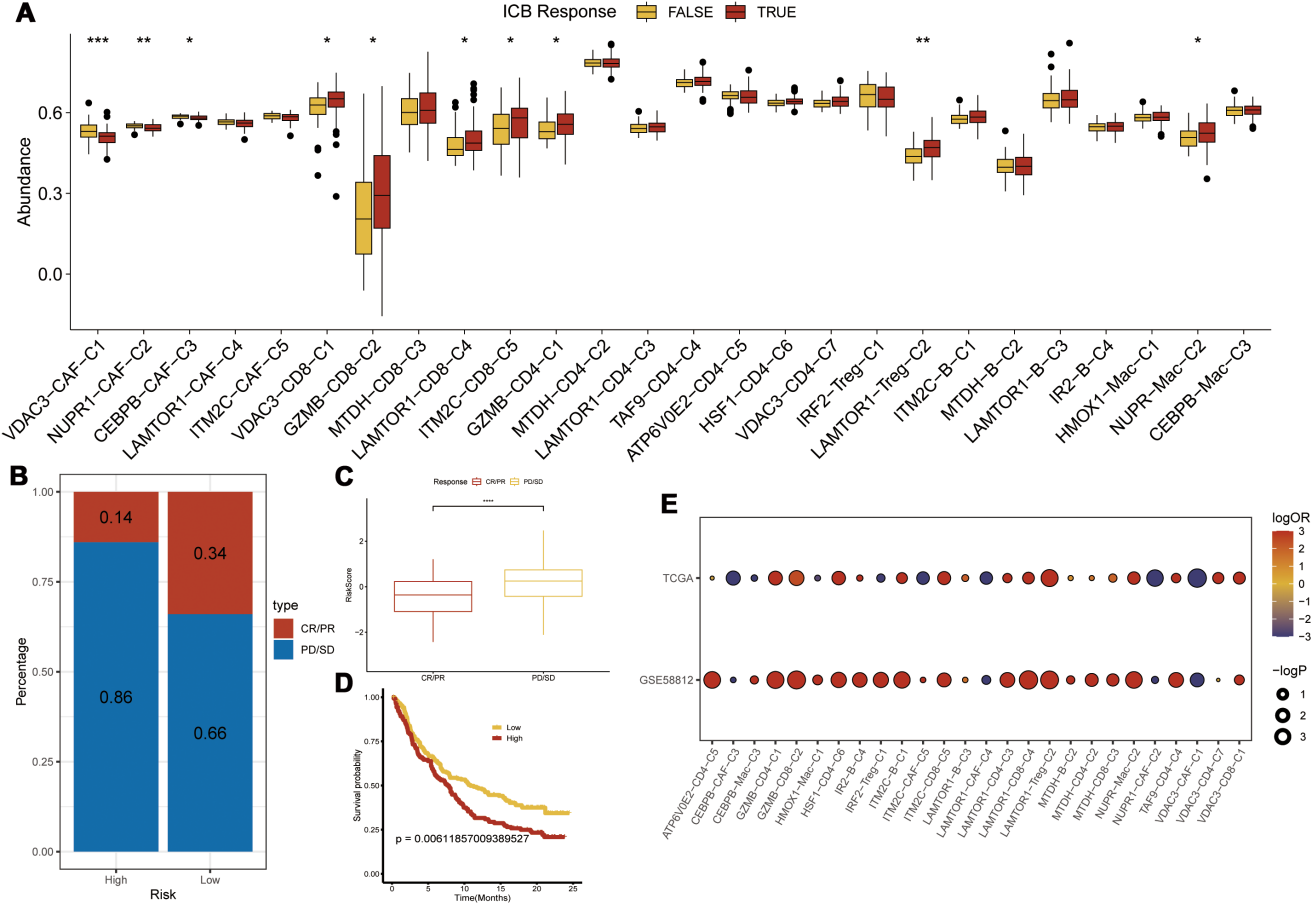
Immunotherapy Prediction. **(A)** Differences in immunotherapy scores among various clusters; **(B, C)** Prediction of immunotherapy response in the high-risk group from the IMvigor210 dataset; **(D)** Prognostic analysis using the risk model in the IMvigor210 dataset; **(E)** Validation of immunotherapy scores in the GSE58812 dataset; Statistical significance is indicated as follows: *p<0.05, **p<0.01, ***p<0.001.

To further characterize the immunotherapy outcomes between the high- and low-risk groups, we performed Tumor Immune Dysfunction and Exclusion (TIDE) analysis to compare the immunotherapy responses across different clusters. Patients in the high CEBPB-CAF-C3 group showed significantly poorer responses to immunotherapy compared to other groups (**Figure 9A**). This demonstrated functional disparities of various genes within distinct cell types. Subsequently, we used the IMvigor210 dataset to validate the efficacy of the prognostic model in predicting immunotherapy outcomes. The high-risk group in the IMvigor210 dataset was associated with a significantly lower response rate and poorer therapeutic outcomes than the low-risk group (**Figures 9B, C**). Furthermore, patients in the high-risk group from the IMvigor210 dataset showed significantly worse prognosis (p<0.001) based on the Kaplan-Meier survival analysis and the log-rank test (**Figure 9D**). This demonstrated the broad clinical applicability of the prognostic model. Finally, we performed a TIDE analysis of data from the GSE58812 dataset to validate the immunotherapy prognosis and showed that the CEBPB-CAF-C3 group was associated with a poorer prognosis than other groups (**Figure 9E**). This further substantiated the immunosuppressive role of CEBPB-CAF-C3 in TNBC.

### 3.5 PCD-regulated CEBPB^+^ CAF subtype is a key determinant of TNBC progression

**Figure 10:**
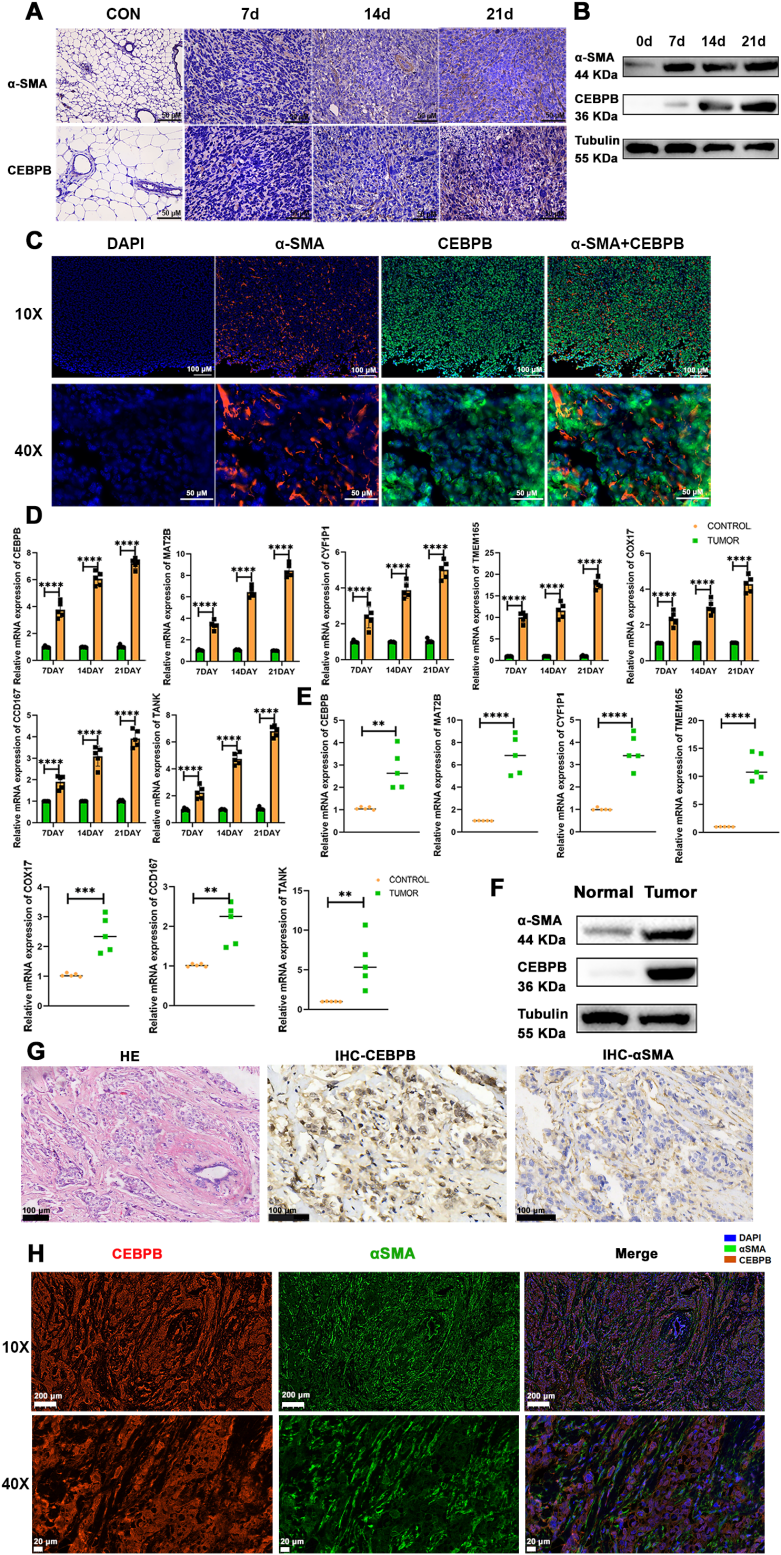
The CEBPB+CAF subtype exists in TNBC and is closely related to tumor progression. **(A)** Immunohistochemical staining to detect the expression differences of α-SMA and CEBPB proteins in normal mouse mammary tissues and TNBC orthotopic tumor-bearing mouse mammary tissues at 7, 14, and 21 days; scale bar: 50 µm; **(B)** Western blot analysis to detect the expression differences of α-SMA and CEBPB proteins in normal mouse mammary tissues and TNBC orthotopic tumor-bearing mouse mammary tissues at 7, 14, and 21 days; **(C)** Multiplex immunofluorescence to detect the expression and localization of α-SMA and CEBPB in mouse mammary cancer tissues; scale bar: 100 µm (whole view), 50 µm (magnified view); **(D)** Quantitative real-time PCR experiment to detect the expression of CEBPB, TANK, MAT2B, TMEM165, CCDC167, CYFIP1, and COX17 in mouse mammary cancer tissues; **(E)** Quantitative real-time PCR experiment to detect the expression of CEBPB, TANK, MAT2B, TMEM165, CCDC167, CYFIP1, and COX17 in human TNBC breast cancer tissues; **(F)** Western blot analysis to detect the expression differences of α-SMA and CEBPB proteins in human TNBC breast cancer tissues and adjacent normal tissues; **(G)** Hematoxylin-eosin (HE) staining showing the structure of human TNBC breast cancer tissues, and immunohistochemical staining to detect the expression differences of α-SMA and CEBPB proteins in human TNBC breast cancer tissues; scale bar: 100 µm; **(H)** Multiplex immunofluorescence to detect the expression and localization of α-SMA and CEBPB in human TNBC breast cancer tissues; scale bar: scale bar: 200 µm (whole view), 20 µm (magnified view). **p < 0.0021, ***p < 0.0002, ****p < 0.0001.

To validate the above findings, we established three different stages of BC progression in a mouse model of TNBC, specifically on days 7, 14, and 21 post-tumor implantation, and assessed the expression levels of the fibroblast marker proteins, α-SMA and CEBPB. The results from immunohistochemistry (IHC) (**Figure 10A**), western blotting (**Figure 10B**), and multiple immunofluorescence (**Figure 10C**) experiments confirmed the presence of the CEBPB+CAF subtype in the TNBC mouse model. Subsequently, we performed quantitative reverse-transcription polymerase chain reaction (qRT-PCR) to examine the expression levels of the seven key prognostic genes—CEBPB, TANK, MAT2B, TMEM165, CCDC167, CYFIP1, and COX17—in the CEBPB+CAF prognostic model in the tumor tissues. The seven key prognostic genes were highly expressed in the murine BC model (**Figure 10D**). This suggested a positive correlation of the prognostic genes with the occurrence of TNBC. We further investigated the CEBPB+ CAF subtype in the TNBC patient tissues and included both cancerous and adjacent normal tissues. QRT-PCR (**Figure 10E**), Western blotting (**Figure 10F**), IHC (**Figure 10G**), and multiple immunofluorescence (**Figure 10H**) experiments confirmed the high expression of the CEBPB+ CAF subtype in the BC tissues.

These results demonstrated the presence of the CEBPB+ CAF subtype in TNBC and its association with tumor progression. Therefore, the CEBPB+CAF subtype may serve as a valuable prognostic predictor for TNBC.

## 4 Discussion

PCD is associated with the progression and treatment of various human cancers. The different forms of PCD not only determine the fate of tumor cells but also significantly influence the dynamic balance in the tumor microenvironment (TME), immune evasion, and the treatment response (27). Previous studies have focused on the disruption of the balance in the TME by different modes of PCD. In this study, we systematically investigated the association of PCD-related prognostic genes with different tumor cell subtypes and the status of the TME in TNBC. In this investigation, we used the single-sample GSEA (ssGSEA) and ImmuneCellAI computational methods to quantitatively assess differences in the cellular compositions between TNBC and adjacent non-neoplastic mammary tissues. Our analysis demonstrated statistically significant alterations in the TME, specifically in the proportions of α-smooth muscle actin-positive (α-SMA+) CAFs, CD68+ tumor-associated macrophages (TAMs), CD19+ B lymphocytes, and distinct T lymphocyte subpopulations (CD4+, CD8+, and FOXP3+ regulatory T cells). Multiparametric analyses of the PCD-associated phenotypic signatures across distinct cell populations demonstrated that the CCAAT/enhancer-binding protein beta-positive (CEBPB+) CAFs exhibited the strongest negative correlation with the survival outcomes of TNBC. Comprehensive analyses of intercellular signaling networks, immune infiltration patterns, and immunotherapeutic response signatures resulted in the identification of molecular mechanisms through which the CEBPB+ CAFs modulate the immunosuppressive microenvironment and promote resistance against immune checkpoint inhibitor treatment in TNBC. To validate these findings, we performed *ex vivo* analyses of tumor specimens from both TNBC patients and syngeneic murine models by quantitative reverse-transcription PCR (qRT-PCR), immunoblotting, immunohistochemical staining, and multiplexed immunofluorescence co-localization assays, and confirmed differential expression of the PCD-related prognostic genes and the presence of CEBPB+ CAFs. Our results demonstrate that PCD-associated pathways are intricately linked to the immunological landscape of TNBC and specific tumor cell phenotypes function as critical modulators of the immune response and therapeutic resistance. These findings suggest that PCD-related mechanisms regulate TME composition and immunotherapy efficacy and have significant implications for disease progression and clinical outcomes in patients with TNBC.

Previous studies have demonstrated the pivotal role of PCD pathways in neoplastic progression and targeted therapeutic interventions through modulation of the TME (28). Through comprehensive transcriptomic analyses, we identified 37 PCD-associated prognostic gene signatures that were significantly upregulated across diverse cellular subpopulations within the TME, thereby establishing a robust correlation between PCD-mediated processes and the architectural and functional dynamics of the TNBC microenvironment. Previous investigations have demonstrated that the TAMs function as primary immunological effector cells that orchestrate diverse immune responses within the TME and as critical modulators of tumor progression and immunosuppressive mechanisms (29). Single-cell RNA sequencing analysis in this study demonstrated differential expression patterns of the PCD-related genes across four distinct TAM subpopulations, each of which demonstrated unique transcriptional profiles and functional characteristics. These findings suggested that the PCD-associated pathways functioned as primary determinants of macrophage phenotypic plasticity within the TME. Beyond the myeloid compartment, variations in the PCD-related signaling cascades contributed to distinct phenotypic and functional heterogeneity among the diverse lymphoid populations within the TME. Furthermore, tumor-infiltrating T lymphocytes exhibited dichotomous phenotypes: (1) an exhausted state characterized by elevated inhibitory receptor expression associated with immunosuppression and poor clinical outcomes; and (2) a cytotoxic phenotype characterized by enhanced effector functions that augment anti-tumor immunity and immunotherapeutic responses (30, 31). Our computational analyses demonstrated that the immune cell clusters associated with T cell exhaustion signatures exhibited high transcriptional activity and enhanced malignant epithelial cell communication networks, whereas the clusters characterized by cytotoxic T cell signatures displayed attenuated transcriptional profiles. This phenomenon corresponded to established mechanisms that dysregulate the TME and contribute to TNBC progression and therapeutic resistance. We identified a distinct cellular subset, designated as GZMB-CD4-C1, which exhibited significant correlation with both exhausted T cells and cytotoxic T cell signatures while maintaining high transcriptional activity. Granzyme B (GZMB), encoded by the GZMB locus is a crucial cytolytic effector molecule in T cell-mediated cytotoxicity. Li et al. demonstrated that the human type 2 innate lymphoid cells (ILC2s) secreted GZMB, which facilitated direct tumor cell lysis through the induction of PCD mechanisms such as pyroptosis and apoptosis (32). The GZMB-CD4-C1 cellular phenotype is characterized by GZMB as a key regulatory gene and exhibits both exhaustion and cytotoxic characteristics. It also potentially represents a critical cellular subset that regulates immune dysregulation in the of TNBC. The dual functionality of the GZMB-CD4-C1 cellular phenotype is potentially regulated by the PCD-associated pathways, but further investigations are necessary to determine the underlying molecular mechanisms.

PCD modulates the phenotypic and functional characteristics of tumor-associated fibroblasts within the tumor microenvironment via multiple molecular mechanisms. Single-cell RNA sequencing analysis in this study identified six distinct CAF subpopulations. Among these, the CEBPB-expressing CAFs (CEBPB-CAF-C3) occupied a central position in the pseudotemporal developmental trajectory. These CEBPB-CAF-C3 cells exhibited upregulated expression of genes associated with proinflammatory cytokine signaling, ECM reorganization, and neoplastic progression. The CEBPB locus encodes the pleiotropic transcription factor CCAAT/enhancer-binding protein beta (C/EBPβ), which functions as a master regulator of cellular bioenergetics, immunomodulation, and inflammatory cascades, and is implicated in the pathogenesis of numerous malignancies (33). Recent molecular studies have reported that aberrant tumor glycolysis regulates the CEBPB signaling axis and modulates the tumor-associated Myeloid-Derived Suppressor Cells through interconnected autophagy and pyroptosis pathways, thereby maintaining the immunosuppressive phenotype within the TME (34). However, the cell type-specific regulatory role of CEBPB in tumor development and progression and immunosuppression in the TME remains to be fully elucidated. Computational analyses using GSVA and multivariate Cox proportional hazards modeling demonstrated that the differential expression patterns of the CEBPB-CAF-C3 cells exhibited superior prognostic value for the TNBC patients compared to other cellular constituents in the TME. Furthermore, we used the *in vivo* syngeneic orthotopic murine models and clinical specimens to verify the presence of the CEBPB+ CAF subpopulation and the increased expression of its signature genes in TNBC using RT-qPCR, immunoblotting, immunohistochemistry, and multiplex immunofluorescence microscopy. This is the first investigation to demonstrate the regulation of elevated CEBPB expression in the CAFs by the PCD-dependent molecular pathways. Therefore, we identified a critical phenotypic determinant associated with poor clinical outcomes in TNBC.

CAFs demonstrate heterogeneous immunomodulatory properties. They exhibit dual functionality including chemokine/cytokine-mediated recruitment of adaptive and innate immune cell populations to TME as well as immunosuppression by promoting M2-like macrophage polarization and attenuating dendritic cell (DC)-mediated antigen presentation and T cell activation (3). Based on the analysis using the TIDE algorithm, patients with elevated CEBPB-CAF-C3 signatures demonstrated significantly reduced responses to immune checkpoint blockade therapy, thereby confirming the immunosuppressive phenotype of this distinct CAF subpopulation. The CEBPB-CAF-C3 cells orchestrated TME remodeling by secreting immunomodulatory cytokines through activation of autocrine Signal Transducer and Activator of Transcription (STAT) and Tumor Necrosis Factor-alpha (TNF-α) signaling cascades. Simultaneously, CEBPB-CAF-C3 cells also regulate the infiltration dynamics and functional heterogeneity of the B lymphocytes, T lymphocytes, and myeloid-lineage cells through the Midkine (MDK)-Nucleolin (NCL) signaling axis. Dose-dependent upregulation of intratumoral MDK in response to gemcitabine administration decreases chemotherapeutic efficacy and enhances neoplastic progression and therapeutic resistance (35). Membrane-associated NCL is a critical mediator of chemotherapeutic resistance because it functions as a pleiotropic regulator of multiple oncogenic processes, including epithelial-mesenchymal transition, anti-apoptotic protein stabilization, and pathological angiogenesis/lymphangiogenesis (36). However, the molecular mechanisms through which CAFs modulate immune cell function via MDK signaling is not fully characterized, and the functional interaction with NCL requires further investigation. Our study provides the first experimental evidence for CEBPB-overexpressing CAFs orchestrating immune cell function through the MDK-NCL signaling axis, thereby promoting TME immunosuppression. Therefore, it represents a potential therapeutic target for overcoming resistance to immune checkpoint blockade therapy.

This study has several limitations. First, further experimental validation through clinical and functional assays is necessary for the distinct immunophenotypic profiles and functional characteristics identified in this study for the tumor-infiltrating myeloid cells, CD4+ and CD8+ T lymphocyte subsets, and B lymphocyte populations. Second, the intricate molecular mechanisms through which CEBPB-CAF-C3 orchestrate TME dysregulation, including cytokine networks and metabolic reprogramming, require in-depth mechanistic investigation and prospective clinical validation. Furthermore, the technical constraints inherent to single-cell RNA sequencing methodologies such as transcript dropout events and stochastic variation across heterogeneous datasets necessitate careful interpretation. Consequently, further studies are necessary to validate our findings and elucidate the underlying molecular mechanisms.

## 5 Conclusion

**Figure 11:**
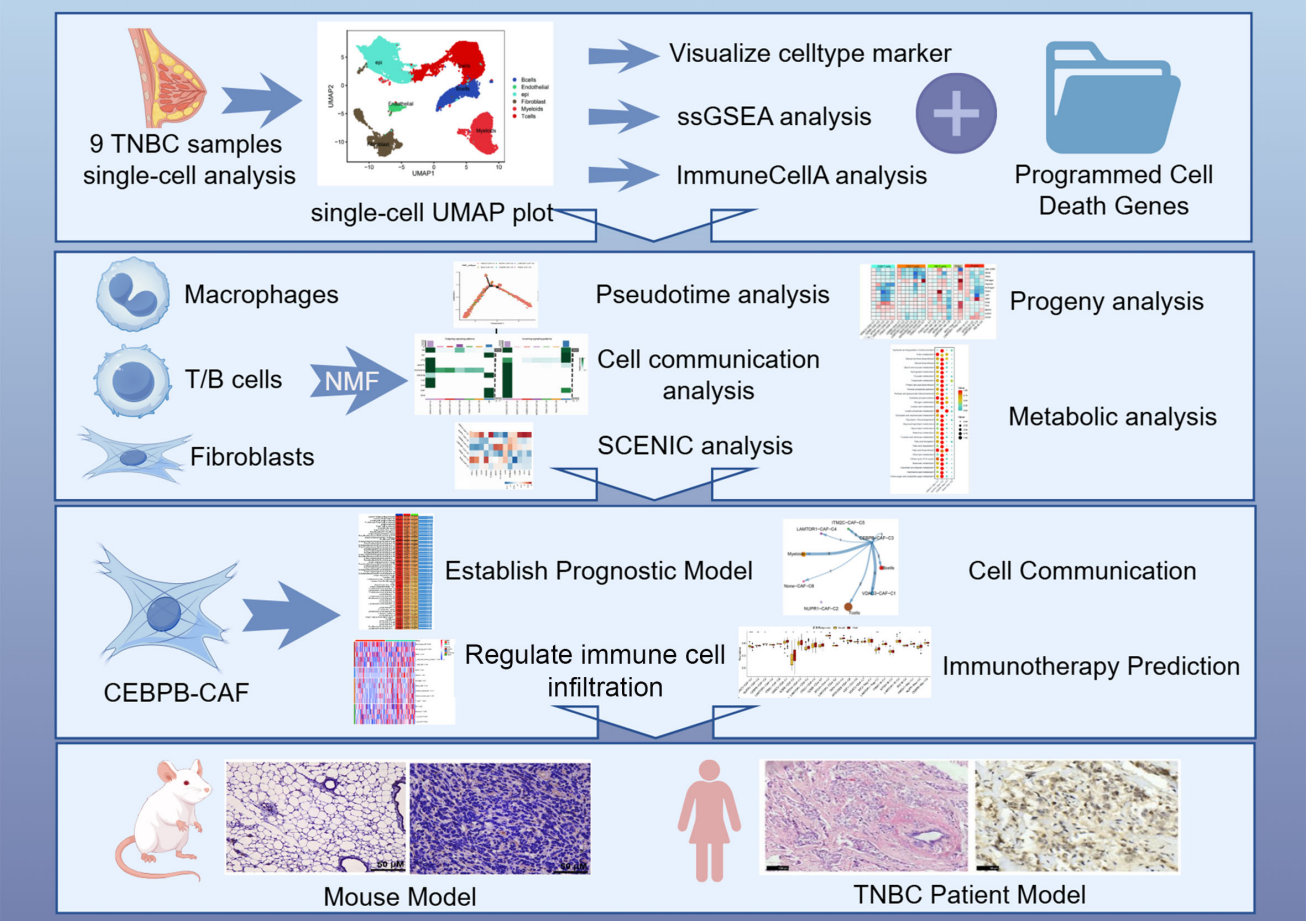
Flowchart of our study process.

In this study, we demonstrated that distinct PCD modalities significantly influenced the cellular and molecular architecture of the TME in TNBC patients through systematic computational analyses of high-dimensional transcriptomic data. We used unbiased clustering algorithms to analyze the immune cell and stromal fibroblast populations and established the regulatory mechanisms through which the PCD-associated gene networks modulate the phenotypic plasticity of the tumor-infiltrating immune cells within the TME. We also identified and validated the existence of a distinct CAF subpopulation characterized by elevated CEBPB expression through multiple orthogonal approaches. Functional characterization of the CEBPB-CAF-C3 cell type demonstrated its crucial role in tumor progression and therapeutic resistance through activation of drug resistance pathways and immunomodulatory mechanisms. Therefore, the CEBPB-CAF-C3 cell type was associated with adverse clinical outcomes in TNBC patients (**Figure 11**). This investigation provides a comprehensive molecular framework for understanding PCD-mediated TME dysregulation in TNBC pathogenesis. Moreover, our study also highlights novel mechanistic insights and potential therapeutic targets for circumventing immunotherapy resistance in TNBC.

## Data Availability

The datasets presented in this study can be found in online repositories. The names of the repository/repositories and accession number(s) can be found in the article/**Supplementary Material**.

## Supplementary material

Supplementary Figure 1 Infercnv predicts vice in epithelial cells.

Supplementary Figure 2 Pseudotemporal analysis of T cell subsets. **(A)** CD8+T cell PCD−related clusters pseudotemporal analysis; **(B)** CD4+T cell PCD−related clusters pseudotemporal analysis;**(C)** NK cell PCD−related clusters pseudotemporal analysis; **(D)** Treg cell PCD−related clusters pseudotemporal analysis.

Supplementary Figure 3 Correlation of key genes with immune score in TCGA. *p<0.05, **p<0.01, ***p<0.001.
