# Supplementary figures and images for: Programmed cell death-related prognostic genes mediate dysregulation of the immune microenvironment in triple-negative breast cancer
Source: Front Immunol. 2025 Mar 12;16:1563630. doi: 10.3389/fimmu.2025.1563630 (PMC11936919; doi:10.3389/fimmu.2025.1563630)

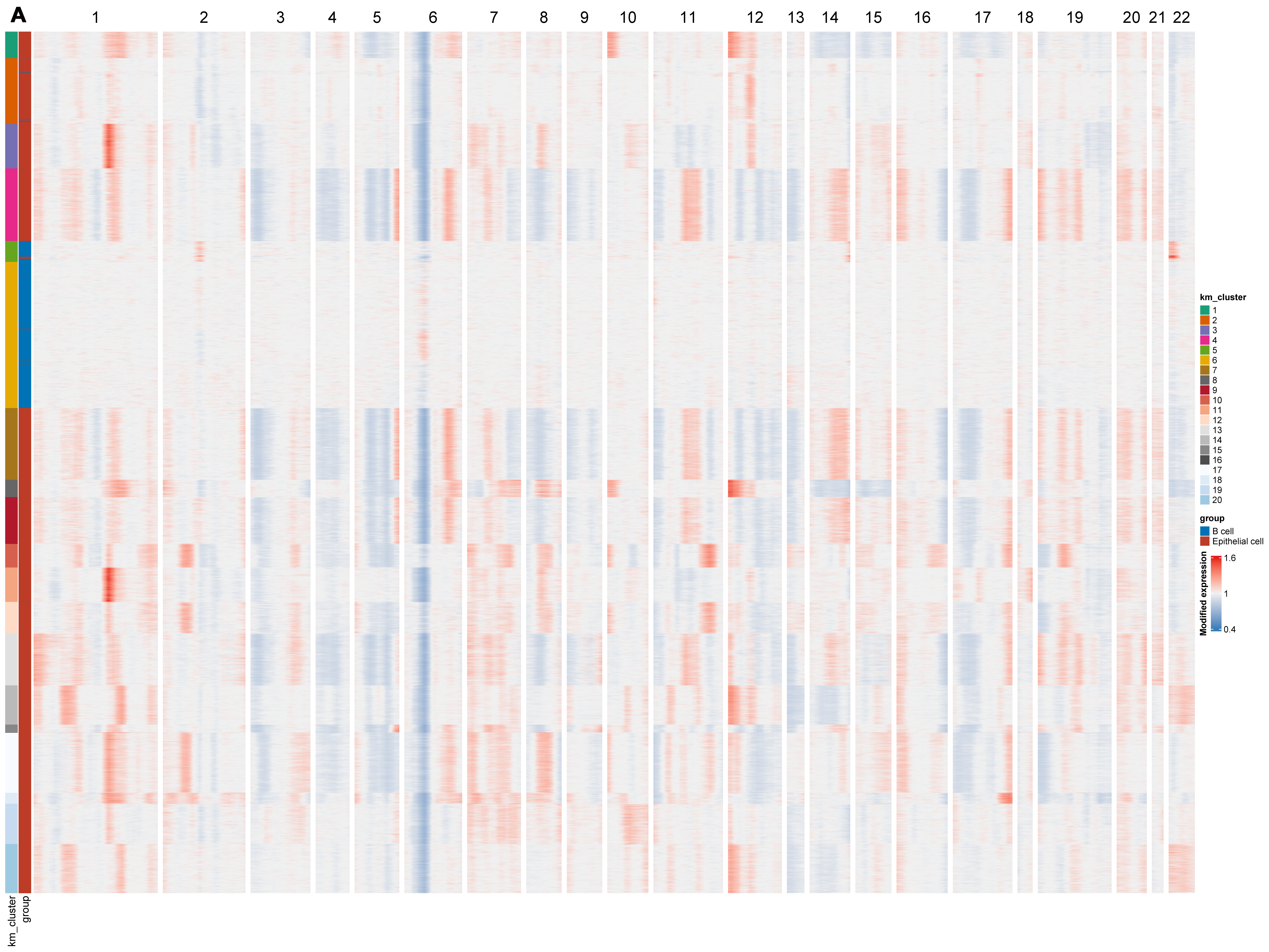

Supplement: Supplementary Figure 1 — Infercnv predicts vice in epithelial cells. [file Image1.tif]

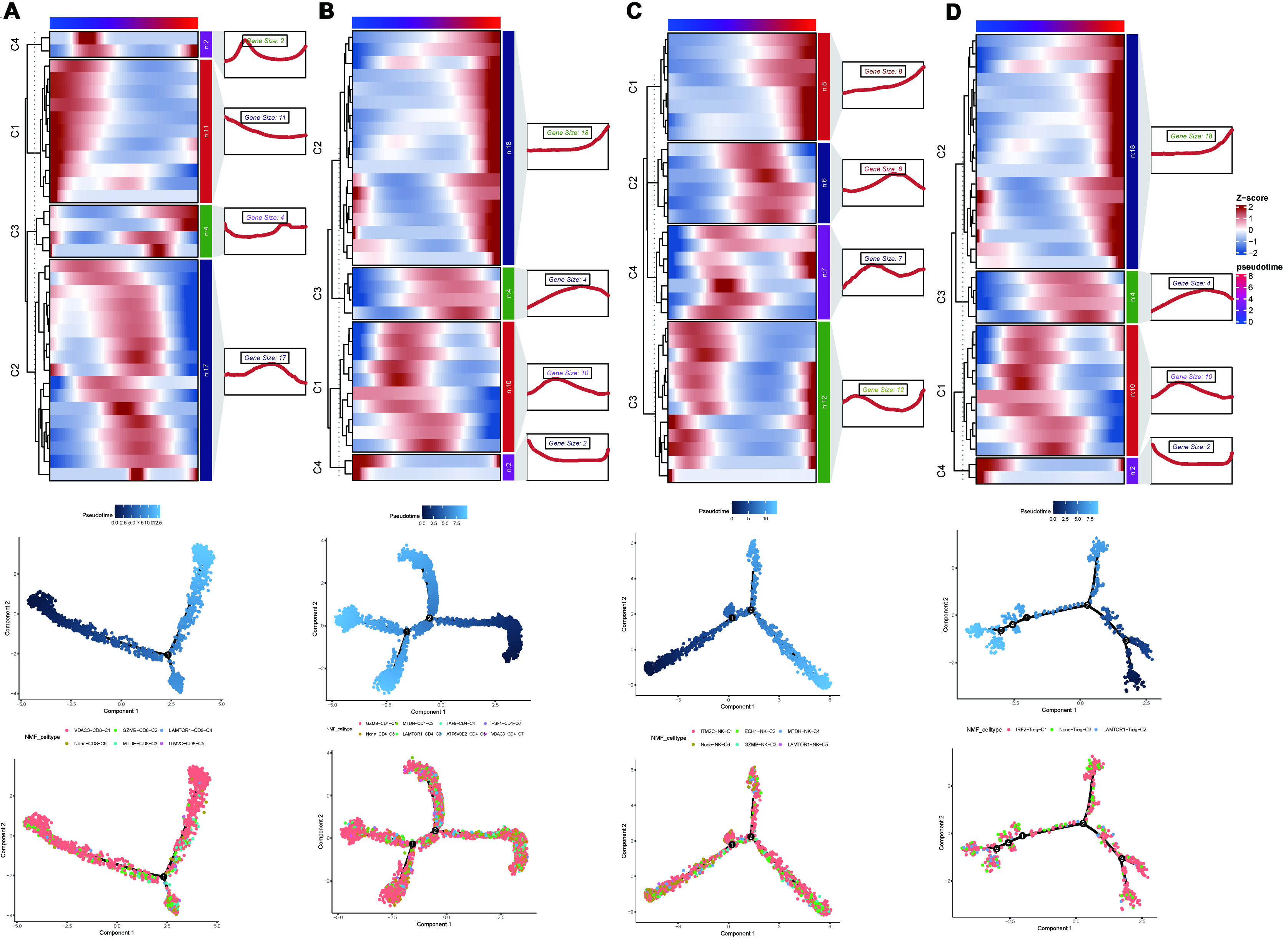

Supplement: Supplementary Figure 2 — Pseudotemporal analysis of T cell subsets. (A) CD8+T cell PCD−related clusters pseudotemporal analysis; (B) CD4+T cell PCD−related clusters pseudotemporal analysis;(C) NK cell PCD−related clusters pseudotemporal analysis; (D) Treg cell PCD−related clusters pseudotemporal analysis. [file Image2.tif]

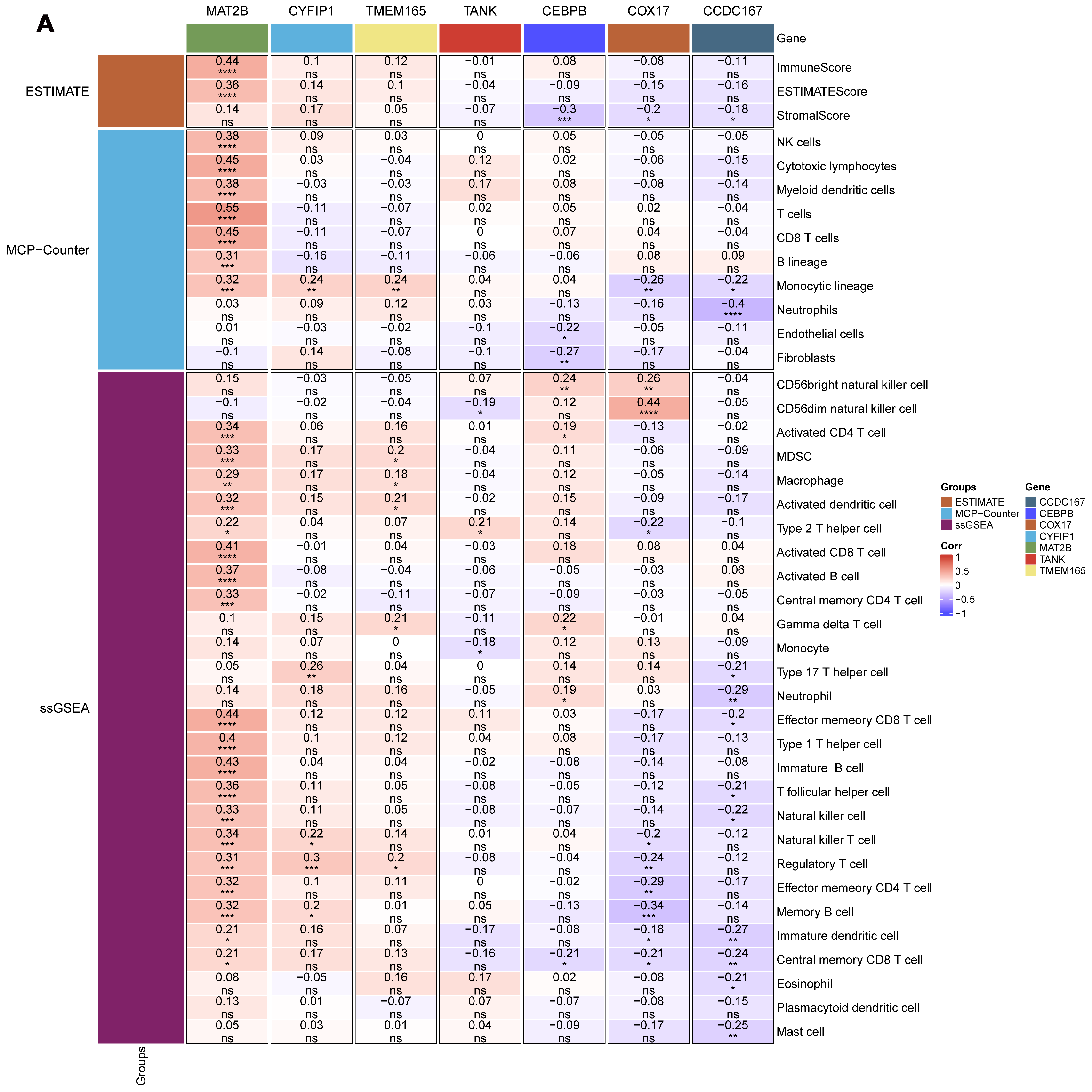

Supplement: Supplementary Figure 3 — Correlation of key genes with immune score in TCGA. *p<0.05, **p<0.01, ***p<0.001. [file Image3.tif]

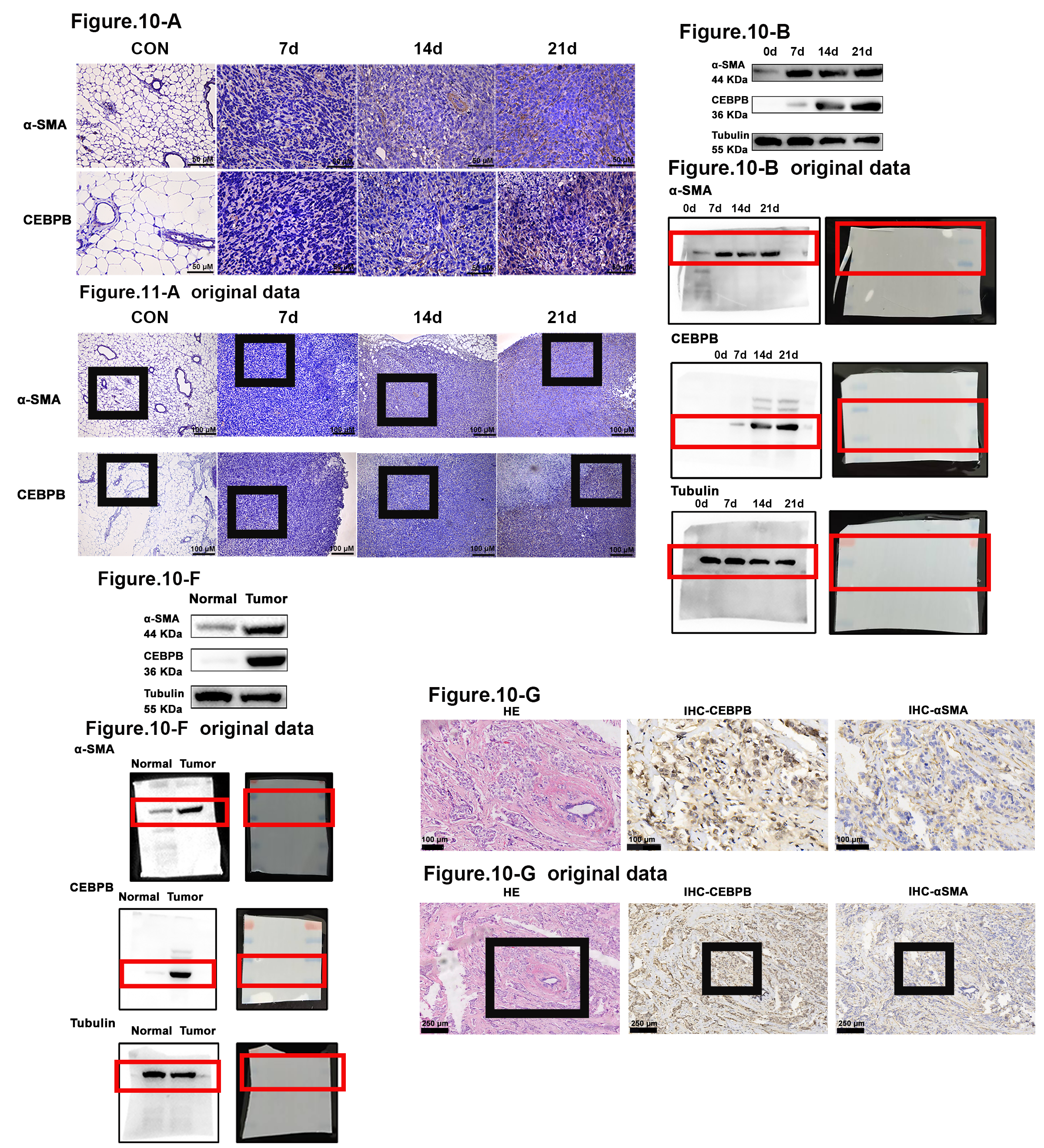

Supplement: Supplementary file 4 [file Image4.tif]
